# Adjustment for day-to-day variability in the estimation of effective concentrations for the assessment of mixture toxicity

**DOI:** 10.1007/s00204-025-04141-w

**Published:** 2025-08-19

**Authors:** Franziska Kappenberg, Tim Brecklinghaus, Leonie Schürmeyer, Wiebke Albrecht, Kirsten Schorning, Jan G. Hengstler, Jörg Rahnenführer

**Affiliations:** 1 Department of Statistics, TU Dortmund University, Vogelpothsweg 87, 44227 Dortmund, Germany; 2 Department of Toxicology, Leibniz Research Centre for Working Environment and Human Factors (IfADo), Ardeystr. 67, Dortmund, 44139 Germany; 3 Institute of Medical Biometry, Informatics and Epidemiology (IMBIE), University Hospital Bonn, Venusberg-Campus 1, 53127 Bonn, Germany

**Keywords:** Mixture toxicity, Interaction, Additivity, Synergism, Concentration–response, Day-to-day variability

## Abstract

Humans are exposed to a multitude of substances at the same time; thus, toxicity assessment for mixtures of several substances and not only for the individual substances is critical. An important challenge in testing the mixture effects of many substances in vitro is the variability between individual experiments, also named ‘day-to-day variability’. Two steps, each consisting of independent experiments, are needed when mixtures are tested. In the first experimental step, for each substance, the concentration–response relationship is usually measured with 6–10 concentrations for at least 3 experiments, and EC_20_ values, i.e., the concentration where a fitted curve intersects with a viability value of 80%, are determined. These values are used as reference values and in the second experimental step, the mixture of several substances is based on these individual EC_20_ values. Since the mixture experiments are new, independent experiments, typically conducted on different days, day-to-day variability can lead to deviations in the cytotoxicity at the reference EC_20_ concentration, i.e., to higher or lower observed viabilities. In this work, a procedure is proposed, how a single concentration per substance tested in the same experiment as the mixture can be used to reliably adjust for day-to-day variability. In the here-established procedure, a specific additive model denoted as ‘budget approach’ is introduced as a reference model to explore potential positive or negative interaction effects between substances.

**Supplementary Information:**

The online version contains supplementary material available at 10.1007/s00204-025-04141-w.

## Introduction

Toxicological risk assessment is mainly based on the characterization of individual substances, while the effects of mixtures are only addressed in specific cases (Braeuning et al 2022). However, considering the increasing number of chemicals to which consumers are exposed, the field of research on potential mixture toxicity has gained attention (Tralau et al 2021; Karaca et al 2023). Concepts of mixture toxicity describe approximately additive effects, for co-exposure to several substances with similar pharmacokinetics acting by a similar mechanism, also named the ‘concept of the multi-headed dragon’ (Bloch et al 2023). On the other hand, combinations of specific substances may lead to more than additive effects, when a second substance enhances the toxic effect of another, named ‘synergy of evil’ (Bloch et al 2023).

Analysis of the effects of mixtures requires reliable statistical methods to calculate if a combination of substances shows additive or more than additive (positive interaction) / less than additive (negative interaction) effects. In particular, a reference model for additive effects is required to study if a combination of substances acts with some positive or negative interaction. The effect of a combination of substances is then measured and compared to the additive reference model. Positive or negative interactions are present if the observed effect is larger or smaller, respectively, than in the additive model (Lee et al 2007). Three additivity models are commonly used, namely 1) effect addition, 2) Bliss independence, and 3) Loewe additivity.

In the effect addition model, the effect of a combination of two substances evaluated at doses $d_1$ and $d_2$, respectively, corresponds to the sum of the individual effects. This model has obvious limitations in scenarios where a sum of two effects becomes larger than technically possible, e.g., when considering percentages since these cannot be larger than 100%. Still, the sum of two individual effects might add up to a value higher than 100% (Lee et al 2007).

The Bliss independence describes the effect size of combining two substances by multiplying their individual effects. These individual effects are expressed as fractions of the maximum effect that would be observed with an infinite dosage of each substance (Bliss 1939).

Three scenarios are identified: First, the two substances are assumed to act independently, which allows the derivation of the combined effect from the individual effects. Second, a similar but still independent action of the substances is assumed, and third, a synergistic effect of the two substances is assumed.

In the Loewe additivity framework, the combination of two substances is assessed on the so-called *isobole* of the doses (Loewe 1927). When considering two axes of doses for the two substances, with the respective dose for a pre-specified effect level marked on each axis, the isobole is the straight line connecting these doses. In mathematical terms, for a pre-specified effect level *y*, and doses *A* and *B* corresponding to the effect level *y* for the two substances, the isobole is defined by

$$\begin{aligned} \frac{a}{A} + \frac{b}{B} = 1, \end{aligned}$$

with *a* and *b* denoting the doses of the two substances in the mixture (Tallarida 2011). The term $\frac{a}{A} + \frac{b}{B}$ is also denoted as *interaction index*. The interaction index of tested mixtures of substances is then compared to the value of 1. Results $<1$ correspond to a positive interaction effect of the substances, and results $>1$ correspond to a negative interaction effect of the substances. These linear isoboles can also be extended to other shapes. A detailed discussion of different types of curved isoboles together with extensive examples is given in Tallarida (2016).

A discussion of the advantages and disadvantages of the Bliss and the Loewe additivity is given in Vakil and Trappe (2019). In addition to this discussion, the article provides an overview of computational models to identify drugs with beneficial effects in a therapeutic setting when administered together. An extensive review of potential synergistic interactions between substances from the application areas of pesticides, metal, and antifoulants (i.e., in the context of ecotoxicology) is presented by Cedergreen (2014). The author concluded that synergies occur only rarely, and instead, additive models play the most important role in assessing the risk of mixtures of substances.

The previously introduced methods mostly focus on a small number of substances and a potential benefit concerning therapeutic uses of a combination of drugs. However, with respect to the vast number of substances humans are exposed to in daily life, specifically a combination of many substances in potentially very low doses is of interest. A new hypothesis of interaction that takes very large numbers of substances into account was presented by Bloch et al (2023) under the name ‘Revolting Dwarfs’. This concept entails a large number of substances, all below individual levels at which negative effects are expected, but due to their large number, some adverse effects are hypothesized. These adverse effects can be caused by sub-additive, additive or synergistic effects. A basis for this hypothesis is given, for example, in Dinca et al (2023), where the authors tested a mixture of 13 substances in rats and came to the conclusion that adverse effects were observed after administration of the substances over a long period and at very low doses.

To obtain information about the cytotoxicity of individual substances, the viability of cells is often measured concentration-dependently for 6-10 concentrations using specific in vitro toxicity assays. Then, a parametric concentration–response curve can be fitted to the resulting viability values. A commonly used model class are log-logistic models, with a sigmoidal, monotonous shape (Ritz et al 2019; Holland-Letz and Kopp-Schneider 2021). So-called ‘alerts’, i.e., concentrations where a pre-specified effect size is attained, can then be calculated. A common type of alert is given by effective concentrations (EC). The EC_20_, e.g., corresponds to the concentration where a fitted curve attains a viability value of $80\%$ (Ritz et al 2019). Other alerts include benchmark concentration estimation (Jensen et al 2019), no observed adverse effects levels (Dorato and Engelhardt 2005), and several alerts specifically tailored to other types of concentration–response data, e.g., where the response is given by gene expression (Kappenberg et al 2021; Möllenhoff et al 2022).

In this work, a theoretical approach for assessing the interaction between a potentially large number of substances based on fixed-proportion mixture response curves is proposed. A mixture of substances is created by combining their individual EC_20_ values according to fixed proportions, e.g., 0.1 times the EC_20_ values, using the same proportion for each substance. The corresponding viability of cells is measured for several proportions and a parametric curve is fitted, where the predictor (the x-axis) is given by the proportion, and the resulting viability gives the response (the y-axis). Here the EC_20_ value is chosen, since it is more robust than the EC_10_, but has fewer solubility issues than the also commonly used EC_50_. The additive model used here to assess the mixture for positive or negative interaction fits into the framework of Loewe additivity, however, with a larger number of substances, and here it will be denoted as *budget approach*.

For assessing the toxicity of mixtures, data of individual substances and of combined exposures are required. Ideally, based on an optimal experimental design, the experiments for individual substances in several concentrations and their combinations (i.e., different ‘conditions’) would be conducted in one batch. A class of parametric models to describe the behavior of, e.g., two substances, measured individually and in combination, is given by surface models, which can be fitted to this type of data (Twarog et al 2021). Such models have the advantage that they describe the complete interaction between two, or in extended versions three, substances, without relying on interaction indices only. However, in cases where the mixtures consist of a large number of substances, it is not feasible to assess all conditions required for this design on a single day. Instead, one approach to substantially reduce the number of conditions is to use toxicity data of individual substances from historical experiments as the basis for the mixture experiments, and the mixture effect is assessed based on experiments, where different ratios of the individual toxicity indicators are used.

A particular challenge for modeling mixture effects is the variability between independent experiments. Typically, mixtures of several substances are based on substance-wise individual toxicity indicators, such as the EC_20_. Thus, two experimental steps are required for measuring and fitting mixture curves. The individual substances are tested in a first set of independent experiments (at least 3) to determine EC_20_ values. Estimation of the EC_20_ values depends on fitting a parametric curve for each of the independent experiments and then choosing the median value. These curves are further denoted as ‘reference curves’. In the second experimental step, mixtures of the substances are tested based on different proportions of the EC_20_ values. Since these two steps consist of individual experiments, the resulting measurements are subject to experiment-to-experiment variability. Often, independent experiments are performed on different days and cytotoxicity may show a substantial day-to-day variability. Typically, the variability from day to day is much higher compared to the variability between technical replicates of the same day. Even though specific protocols and standardized procedures are used to increase reproducibility, some variation is unavoidable. This may occur due to environmental reasons like differences in temperature and humidity, or the person performing the experiments, but also technical reasons like variation in instrument performance or cell physiology (Petersen et al 2019; Larsson et al 2020). In this paper, all variability in cytotoxicity in the independent experiments is further denoted as ‘day-to-day variability’.

An often observed pattern is that cytotoxicity, here quantified by EC_20_ values, may be higher (or lower) for the tested substances in the experiment in the first step compared to the independent experiment in the second step. If one would interpret the result of the mixture experiment in the second step based on the assumed individual toxicities of the individual substances, without taking the day-to-day variability into account, this would lead to an over- or underestimation of the mixture effect. To quantify the extent of the day-to-day variability, in addition to the complete mixture curves, the viability of the reference EC_20_ in the same experiment as conducted for the mixture curves is determined. The resulting value is also described as ‘day-specific viability’, and the effect is sometimes equivalently referred to as ‘day-specific cytotoxicity’. This allows for an adjustment procedure to take this day-to-day variability into account when evaluating the mixture curves. Here, a method is proposed to account for the day-to-day variability, influencing the actual toxicity of each of the substances administered to the cells. The basic idea of this method is to consider the reference curve and to back-calculate the concentration leading to the observed day-specific viability.

This paper is structured as follows. In Methods, the estimation of effective concentrations from individual concentration–response curves is briefly introduced, and the general setup of the mixture experiments is explained. The specific budget approach is introduced, as well as the required adjustments due to deviations of cytotoxicity between individual experiments, i.e., the day-to-day variability. Several steps of the overall procedure make use of point estimates of effect sizes or concentrations, all of which have a certain statistical variability. The different sources of variability are briefly listed and discussed. In Application, the method is applied to an exemplary combination of 10 substances and all intermediate steps are presented in detail.

## Methods

The basis for measuring mixture effects is the determination of the reference effective concentration EC_20_, i.e., the concentration that leads to a viability of $80\%$ for each substance. These effective concentrations are then combined for several substances in different proportions to obtain the mixture curve. Here, mixtures of *n* substances are considered. In this chapter, first, the estimation of the individual effective concentration is described followed by a description of how to fit the mixture curves. A basic assumption for the mixtures is that of additive effects. Therefore, this assumption and its implication for the curve fitting (the ‘budget approach’) are described. However, deviations between the originally derived EC_20_ and the day-specific cytotoxicity occur due to day-to-day variability. For this, an adjustment procedure is proposed. Finally, a discussion of the various sources of variation within this procedure is provided.

### Estimation of the reference EC_20_

The CellTiter-Blue (CTB) cell viability assay is a commercialized form of the resazurin reduction assay distributed by promega. Resazurin is a cell-permeable redox indicator which is reduced into fluorescent resorufin by viable cells. Nonviable cells rapidly lose their metabolic capacity and thus do not generate a fluorescent signal. A detailed protocol for the CTB assay is provided in the Supplement. In brief, 15000 HepG2 cells were cultured in 200${\upmu }$l Dulbecco’s Modified Eagle Medium containing 10% fetal bovine serum on collagen-coated black 96-well plates. The cells were incubated the next day with the corresponding substance, mixture and solvent control for 48 h. After three washing steps with 1xphosphate buffered saline, the cells were incubated with medium containing 20% CTB reagent. Finally, fluorescence was measured (Ex540nm/Em595nm) using a fluorescence plate reader.

The individual viability curves are fitted as described before (Albrecht et al 2019; Brecklinghaus et al 2022). In the corresponding plots, the x-axis shows the concentration values, and the y-values the resulting viability values. Data are pre-processed by averaging replicates of the control values for each biological replicate, dividing fluorescence values of all replicates by this number and multiplying by 100 to obtain percentages. A flat model, the sigmoidal and monotonous four-parametric-log-logistic (4pLL) (Ritz et al 2019), and the non-monotonous Brain-Cousens (BC) (Brain and Cousens 1989) model are fitted to the pre-processed data. The model formulas are given in the Supplement. The Akaike information criterion (Akaike 2011) is used to select between the three different models. Data points are re-normalized such that in the case of the flat model, the mean response value corresponds to 100%, and in the case of the 4pLL and the BC model, the left asymptote corresponds to 100% (Kappenberg et al 2020). A goodness-of-fit statistic is calculated as

$$\begin{aligned} 1 - \frac{\text {sum of squared differences between data points and fitted curve}}{\text {sum of squared differences between data points and the mean response}}. \end{aligned}$$

This statistic yields values near 1 for a good fit. The effective concentrations (EC values) can only be calculated for those curves fitted with the 4pLL or the BC model. Further, EC values are calculated only for those curves where the response for the highest tested concentration of the respective substance, $\hbox {conc}_{\max }$, is smaller than 90% after normalization, and where the goodness-of-fit is larger than 0.55 (Albrecht et al 2019). The EC_20_ corresponds to the concentration at which the fitted and re-normalized curve attains a response value of 80%. For curves for which EC_20_ cannot be calculated, due to any of the reasons stated above, or because the curve does not have an intersection with the value $80\%$, the value is imputed by $\hbox {conc}_{\max } \times 5$ to avoid missing values. Results for several curves (individual experiments) per substance are summarized via the median. This yields a concentration $c_i$ per substance *i*, $i=1,..., n$, denoting the median EC_20_ of all curves, which is used for the experiments for generating the mixture curves. These curves are further denoted as ‘reference curves’, and the concentration $c_i$ as ‘reference EC_20_’.

### Fitting of mixture curves

A combination of substances, denoted as ‘mixture set’ or ‘set’, is selected to form a specific mixture. The reference EC_20_ values $c_1, \ldots , c_n$ are used as the basis for the mixture of the substances $1, \ldots , n$. Different proportions of the concentrations $c_i$ are administered to cells for the resulting mixture–proportion–response curves, denoted as mixture curves. A proportion of 1 means that for all substances, the concentration $c_i$ is used, resulting in the mixture $1\cdot \sum _{i=1}^{n}{c_i}$. A proportion of 0.5 means that for all substances, the concentration $0.5 \cdot c_i = \frac{c_i}{2}$ is used, resulting in the mixture $0.5 \cdot \sum _{i=1}^{n}{c_i}$. In general, a proportion *p* means that for all substances, the concentration $p\cdot c_i$ is used, resulting in the mixture $p\cdot \sum _{i=1}^{n}{c_i}$. Depending on the size of the set and on resulting solubility restrictions, viability values for a certain number of different proportions (here: eight), plus a negative control (corresponding to a proportion of 0, i.e., using only the solvent), are measured in four technical replicates per experiment using the CTB assay as described before.

Formally, a mixture curve is based on an experiment where the viability of cells is measured for *d* increasing proportions $p_1< p_2< \ldots < p_d$ of the mixture obtained from the individual reference EC_20_ values $c_i$. Consider a scenario with *n* substances, *N* samples overall of one biological replicate, and *d* increasing proportions plus a negative control. Then, the exact design $\xi _N$ for this biological replicate, for equal number of technical replicates $t:=\frac{N}{d+1}$ per proportion, is defined as

$$\xi _N = \begin{pmatrix} 0 & p_1 & p_2 & \ldots & p_d \\ \frac{t}{N} & \frac{t}{N} & \frac{t}{N} & \ldots & \frac{t}{N}. \end{pmatrix}$$

For mixture proportion $p_j$, the corresponding measured concentration in the mixture is given by $p_j \cdot \sum _{i=1}^{n}{c_i},$ for $j=1, \ldots , d$. The highest proportion $p_d$ may be equal to 1, i.e., considering the mixture of all reference EC_20_ values $c_i$, but especially for a high number of substances, this might lead to solubility issues, and then a proportion lower than 1 is chosen as highest value. This type of design, where a mixture of several substances at fixed mixing ratios but with increasing total concentration is also called ‘ray design’ (e.g., Meadows et al 2002).

The selection of substances for the set is based on the previously fitted individual viability curves for a large number of substances. It is required that the EC_50_, i.e., the concentration at which the fitted curve attains a value of 50%, can be calculated for the substances, to ensure the presence of clear cytotoxicity. Solubility of the substances in the medium at a concentration of 10 times the EC_20_, or in the solvent at a concentration of 1000 times the EC_20_ is required as well to ensure a sufficiently high final concentration of the test substance while maintaining the maximum tolerable solvent concentration.

The resulting fluorescence values as obtained from the CTB assay for the mixture curves are pre-processed as described above, i.e., control values for one experiment are averaged and all other fluorescence values are divided by this value and multiplied by 100. The 4pLL model is fitted to the normalized response values, and data are re-normalized such that the upper asymptote corresponds to a value of 100%. Thus, the predictor variable (the x-axis) for the curves consists of the different proportions and the outcome variable (the y-axis) of the resulting normalized viability.

### Budget approach

To analyze mixtures for potential positive (more than additive) or negative (less than additive) interaction effects, the basic assumption is made that no such effects are present, and instead, the substances act in a *purely additive way*. This assumption entails the idea of a *toxicity budget*: For a single substance, in this context, the target alert EC_20_ with corresponding viability of $80\%$ is assumed to correspond to a budget of $100\%$. Within the budget framework and under the assumption of a purely additive effect, in the easiest case of only two substances, it is assumed that applying a proportion $(100 - k)\%$ of the reference EC_20_ for substance 1, i.e., of $c_1$, and a proportion of $k\%$ of the reference EC_20_ for substance 2, i.e., $c_2$, to the cells, will again lead to a viability of $80\%$. This motivates the term budget: It refers to the overall cytotoxicity that (here, considering the EC_20_) results in a $20\%$ reduction in cell viability. This level of cytotoxicity can be achieved either by administering a single substance or using an equivalent mixture of several substances. In both cases, the combined toxicity corresponds to a specific budget that leads to the same decrease in cell viability.

This concept is also the basic assumption of the Loewe additivity (Loewe 1927). In the application considered here, a series of equal proportions of the reference EC_20_ values $c_i$ are considered, and a mixture–response curve is fitted to the data. So in the case of two substances, it is assumed that for a proportion of 0.5 of the values $c_1$ and $c_2$, the curve intersects with a value of $80\%$ viability. In the general case, for *n* substances, under the assumption of purely additive effects, for a proportion of $\frac{1}{n}$ of the respective reference EC_20_ values $c_i, i = 1, \ldots , n$, the curve is expected to intersect with a response value of $80\%$.

**Fig. 1:**
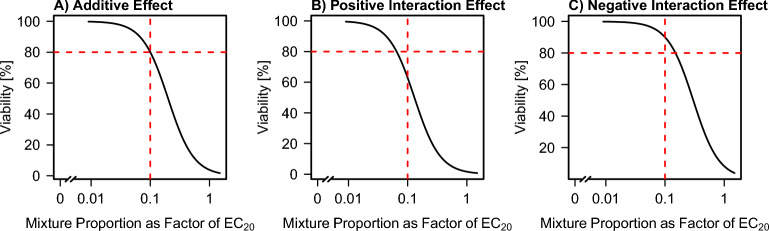
Exemplary display of **A** an additive effect, **B** a positive (i.e., more than additive) and **C**) a negative (i.e., less than additive) interaction effect. For all curves, corresponding to mixtures of 10 substances, the x-axis displays the proportion of the reference EC_20_ value per substance used in the mixture. The mixture proportion of 0 corresponds to administering the medium to the cells only, i.e., to a negative control. Curves are evaluated at a proportion of $\frac{1}{10} = 0.1$, and the response (i.e., the viability) is compared with a value of $80\%$ (red dashed lines). **A** For an additive effect, the response at proportion $\frac{1}{10}$ corresponds to $80\%$. **B** For a positive interaction effect, the response at proportion $\frac{1}{10}$ is below $80\%$. **C** For a negative interaction effect, the response at proportion $\frac{1}{10}$ is above $80\%$

A hypothetical mixture curve that perfectly adheres to this assumption of purely additive effects is displayed in Fig. 1A. Here, a mixture of 10 substances is assumed, so at the proportion of $\frac{1}{10} = 0.1$, the mixture curve intersects with the viability value of $80\%$.

In the case of positive interaction effects, the observed viability of a set of substances, evaluated at the proportion $\frac{1}{n}$ should have a higher toxic effect, i.e., lead to a viability below $80\%$. Such a situation is shown in Fig. 1B. In contrast, in the situation of negative interaction effects, the toxic effect of the combination of the substances should be lower, resulting in a viability above $80\%$ at the proportion $\frac{1}{n}$. This situation is shown in Fig. 1C.

Note that during all steps in this procedure, only point estimates of the respective measures are used, without taking the variability into account. This is further expanded on in Sect. 2.5. Thus, instead of comparing the predicted viability to a value of exactly $80\%$, an interval of $[75\%, 85\%]$ should be considered, for which additivity is inferred if the predicted viability lies within this interval. Consequently, viabilities above $85\%$ correspond to negative interactions and viabilities below $75\%$ to positive interactions.

The approach proposed here for analyzing mixture toxicity is called *budget approach*, since it is based on the assumption that, in the additive approach, organisms (here: cells) can handle a certain amount, denoted as budget, of toxicity, regardless of whether it is administered via one or several substances. Combining a proportion of $\frac{1}{n}$ of all individual reference EC_20_ values $c_i$ of *n* substances is assumed to lead to the same effect as if only one substance is considered, with its individual full reference EC_20_ value (i.e., a proportion of $1\cdot c_i$). Consequently, a budget of $100\%$ corresponds to the full effect as observed at the individual EC_20_ of one substance, i.e., to a response of $80\%$ in terms of viability. It is important not to confuse the intended percentage of $100\%$, corresponding to the budget, with the percentage of $80\%$, corresponding to the response value in terms of viability.

To give an overview of the central terminology and notation within this approach, a summary of all keywords and notation is given in Table 1.

**Table 1:** Overview of the relevant keywords and notation for the Budget approach and the adjustment for day-to-day variability

Keyword	Meaning
Budget	Concept of additivity in assessing the cytotoxicity of a mixtures of substances, based on the assumption that the toxicity elicited by one substance at a specific concentration (here: EC_20_) is the same as the toxicity elicited by several (here: *n*) substances at corresponding proportions of the concentration (here: $\frac{1}{n}$EC_20_, respectively.)
Budget approach	Approach for evaluating the mixture toxicity of several substances under the assumption that a single substance in a fixed concentration is just as toxic as a mixture of several substances in respective lower concentrations (see ‘Budget’). This allows the evaluation of mixture curves at proportion $\frac{1}{n}$ for *n* substances. The approach fits into the Loewe additivity framework
Day-specific viability/ day-specific cytotoxicity	Viability (or correspondingly the cytotoxicity) when measuring the response value at the reference EC_20_ value $c_i$ on a different day. A value of $80\%$ is expected in a scenario without day-to-day variability
Day-to-day variability	Variability between experiments conducted on different days. This refers particularly to variability between the reference and the mixture curves
Median approach	Approach for determining the value ${\tilde{c}}_i$, where all independent reference curves are considered, and the median value of individual values ${\tilde{c}}^{(j)}_i$ is used, where each *j* denotes a curve and *i* is the substance
Mixture set	Set of *n* substances considered for the mixture experiment
Reference curves	First experimental step, where for each substance individually, at least three independent concentration–response curves are measured. From these curves, the median EC_20_ value $c_i$ for substance *i* is derived
Responsible approach	Approach for determining the value ${\tilde{c}}_i$, where only the reference curve that was responsible for the median value, i.e., the value $c_i$, is used

Notation	Meaning
$B^{\text {adj}}$	Adjusted budget for the mixture of all substances in the set
$B^{\text {adj}}_i$	Adjusted budget for the *i*-th substance in the set
$c_i$	Reference median EC_20_ value for substance *i*, used as basis for the mixture experiment
${\tilde{c}}_i$	Concentration where the reference curve for substance *i* attains the day-specific viability value ${\tilde{r}}_i$
${\tilde{c}}^{(j)}_i$	Individual values where each of the reference curves *j* for substance *i* attains the day-specific viability value ${\tilde{r}}_i$, used in the Median approach
$i = 1, \ldots , n$	Index of the substances
*n*	Overall number of substances in the mixture
$p_1< p_2< \ldots < p_d$	Mixture proportions, where for proportion $p_j$, the corresponding measured concentration in the mixture is given by $p_j \cdot \sum _{i=1}^{n}{c_i}$, for $j = 1, \ldots , d$
$\text {prop}^{\text {adj}}$	Adjusted proportion where to evaluate the mixture curve in the final step of the overall procedure
${\tilde{r}}_i$	Day-specific viability for substance *i*, i.e., the response value when measuring the viability at concentration $c_i$ on the same day as the mixture experiment

### Adjustment for day-to-day variability

An important issue in performing the experiments for the mixture curves is that due to variability between independent experiments, response values at the reference EC_20_ value $c_i$ do not necessarily correspond to a response value of $80\%$ when repeating the experiment on a different day. Specifically, in addition to the complete mixture curve, the response values at the substance-wise reference EC_20_ value $c_i$ should be measured in several technical replicates at the same time and under the same circumstances as the complete mixture experiment. If, after suitable pre-processing, including the transformation to percentages, the viabilities at reference EC_20_ values $c_i$ in the new experiment do not correspond to $80\%$ for at least one substance *i*, this affects the overall budget administered to the cells. Assuming the day-specific viability ${\tilde{r}}_i$ at concentration $c_i$ for substance *i* is smaller than $80\%$, this means that under the current experimental conditions, the substance has a stronger cytotoxic effect compared to the reference experiment. Thus, also in the mixture, more than the assumed share of the overall budget is applied, leading to an overall higher cytotoxic effect of this substance within the mixture. On the other hand, if the day-specific viability ${\tilde{r}}_i$ is larger than $80\%$, this corresponds to a weaker cytotoxic effect of the substance *i* compared to that observed in the reference experiments.

Since the day-to-day variability may influence the cytotoxic effect of substance *i* at the reference EC_20_ value $c_i$ in either direction, deviations in the budget spent for individual substances within the mixture can occur. Thus, we propose an approach to calculate an adjusted budget for each substance and subsequently determine an adjustment factor, to determine the ‘adjusted proportion’ at which to evaluate the mixture–response curve for the entire set of substances. The budget adjustment is first presented for an individual substance, and then applied to the entire mixture via averaging across all substances in the mixture set. Two variants of this approach are identified.

The entire procedure consists of four steps: The basis of the procedure is given by estimates of the reference median EC_20_ value per substance $c_i$, which were calculated from several, but at least 3, reference curves. The first step is simultaneously measuring the mixture curves (i.e., measuring the viability for increasing proportions of the reference EC_20_ values $c_i$) for the entire set of substances and the day-specific viability ${\tilde{r}}_i$ for each substance in the mixture set. In the second step, the adjusted budget for the mixture of substances is calculated. The third step is then to adjust the proportion where to evaluate the mixture curve, and the final step is the evaluation of the fitted mixture curve at the adjusted proportion and the comparison of the result to $80\%$ (or, due to the variability, to the interval $[75\%, 85\%])$, to identify if there is a positive or negative interaction effect of the substances, as explained in the general budget approach.

Consider a substance *i* with a reference EC_20_ value $c_i$ obtained as the median of the EC_20_ values of several reference curves. Assume that for the day-specific viability at concentration $c_i$ in the new experiment, the mean response value is given by ${\tilde{r}}_i$, which in particular is different from $80\%$. In the simplest case of the procedure, assume that there is only one reference curve, based on which the reference EC_20_ value $c_i$ was determined. The basic assumption made is that the concentration–response profile displayed in the reference curve is representative, and thus the budget is adjusted based on this curve. Based on the reference curve, the concentration ${\tilde{c}}_i$ is determined at which the curve attains the value ${\tilde{r}}_i$. Then, the adjusted budget $B^{\text {adj}}_i$ for substance *i* is given by

1 $$\begin{aligned} B^{\text {adj}}_i = \frac{{\tilde{c}}_i}{c_i} \cdot 100\%. \end{aligned}$$

The intuition behind the adjustment approach in Equation (1) is as follows: If ${\tilde{r}}_i$ is smaller than $80\%$, i.e., when the day-specific cytotoxic effect is stronger than in the reference experiment, the concentration ${\tilde{c}}_i$ is larger than the reference EC_20_ value $c_i$. Thus, since $\frac{{\tilde{c}}_i}{c_i} > 1$, the budget corresponding to substance *i* that is administered to the cells is larger than $100\%$. Vice versa, for a ${\tilde{r}}_i$ larger than $80\%$, it holds ${\tilde{c}}_i < c_i$ and thus $\frac{{\tilde{c}}_i}{c_i} < 1$. This corresponds to a budget of less than $100\%$ exerted by this substance.

**Fig. 2:**
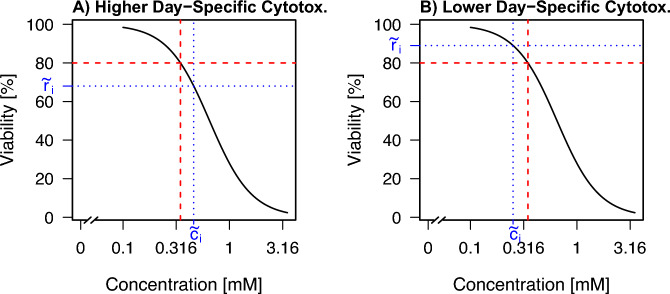
Two examples of day-to-day variability and the resulting concentration ${\tilde{c}}_i$ based on a reference curve. The reference curve is displayed (black line) together with the corresponding value of the reference EC_20_ value $c_i$ (red dashed line). It is assumed that two independent experiments, where the viability is measured at the reference EC_20_ value $c_i$, lead to two different values of ${\tilde{r}}_i$. A) The day-specific cytotoxicity is higher, i.e., ${\tilde{r}}_i < 80$. The corresponding ${\tilde{c}}_i$ (blue dotted lines) is larger than the reference EC_20_ value $c_i$. B) The day-specific cytotoxicity is lower, i.e., ${\tilde{r}}_i > 80$. The corresponding ${\tilde{c}}_i$ (blue dotted lines) is smaller than the reference EC_20_ value $c_i$

This approach is illustrated in Fig. 2 A) for ${\tilde{r}}_i < 80\%$ and in Fig. 2 B) for ${\tilde{r}}_i > 80\%$.

In practice, there is not just one reference curve, but, as described before, the reference EC_20_ value $c_i$ is obtained as a median of EC values estimated from several concentration–response curves. There are two general approaches to consider this:

1. Approach **Responsible:** Consider only the reference curve that is ‘responsible’ for the median of the curve-wise EC_20_ values, i.e., for the concentration $c_i$. *Note:* If an even number of reference curves are available, two of these curves are responsible for the median. In that case, consider the two responsible curves and use the mean of both values for ${\tilde{c}}_i$.
2. Approach **Median:** Consider all *k* reference curves, and determine a value ${\tilde{c}}_i^{(j)}$ for substance *i* for each curve *j* individually, $j = 1, \ldots , k$. Then, use ${\tilde{c}}_i = \underset{j}{\text {median}}({\tilde{c}}_i^{(j)})$.

**Fig. 3:**
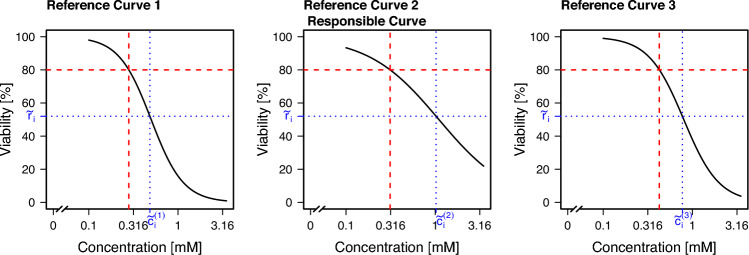
Exemplary display of a situation, where the two approaches **Median** and **Responsible** lead to different values of ${\tilde{c}}_i$. Three reference curves corresponding to one substance *i* are displayed. The EC_20_ values are shown via red dashed lines. The second curve is responsible for the median of the three EC_20_ values. The day-specific viability for that substance (${\tilde{r}}_i$) is lower than $80\%$. The curve-wise concentrations corresponding to that viability are determined and denoted by ${\tilde{c}}_i^{(j)}, j=1,2,3$ (blue dotted lines). The median of these values is given by ${\tilde{c}}_i^{(3)}$, thus, in the **Median** approach, this value would be used, and for the **Responsible** approach, ${\tilde{c}}_i^{(2)}$ would be chosen

An exemplary illustration of these two approaches in the case of three reference curves is shown in Fig. 3. For the three depicted curves, the second one was responsible for the median value of the EC_20_ (red dashed line). This can be seen from the fact that the vertical red dashed line is at the median of the three values for the second curve. However, due to different slopes, when finding the individual values ${\tilde{c}}_i^{(j)}, j=1,2,3$ for a fixed value ${\tilde{r}}_i$, the median of the three resulting values is given by ${\tilde{c}}_i^{(3)}$, the value for the third curve.

While the two approaches presented above differ slightly in terms of how ${\tilde{c}}_i$ is calculated, nevertheless, both result in a—possibly different— calculation of the adjusted budget $B^{\text {adj}}_i$. This procedure is conducted substance-wise, yielding individually adjusted budgets per substance, where some might be above $100\%$ (indicating stronger day-specific cytotoxic effects) and some below $100\%$ (indicating weaker day-specific cytotoxic effects). To determine the adjusted budget for the entire mixture of all *n* substances in the set, the arithmetic mean of the individual budgets is calculated to obtain $B^{\text {adj}}$:

2 $$\begin{aligned} B^{\text {adj}} = \frac{1}{n}\sum _{i=1}^{n}{B^{\text {adj}}_i} \end{aligned}$$

As a specific example, consider a mixture of 10 substances, with individually adjusted budgets taking the values $200\%$, $60\%$, $150\%$, $130\%$, $140\%$, $190\%$, $220\%$, $160\%$, $180\%$, $70\%$. Then, the mean adjusted budget $B^{\text {adj}}$ corresponds to $150\%$.

The final step of the adjustment approach is to determine the concentration at which the mixture curve is evaluated. In a perfect scenario without deviations in the cytotoxicity due to day-to-day variability, for a mixture of *n* substances, the curve would be evaluated at a proportion of $\frac{1}{n}$, and the response value would be compared to $80\%$. In the case of deviations, i.e., mean adjusted budget unequal to $100\%$, the value of $\frac{1}{n}$ must be adjusted as well, and the curve is evaluated at that adjusted proportion, denoted by $\text {prop}^{\text {adj}}$. The value of $\text {prop}^{\text {adj}}$ for the mixture of *n* substances with mean adjusted budget $B^{\text {adj}}$ is given by

3 $$\begin{aligned} \text {prop}^{\text {adj}} = \frac{100}{B^{\text {adj}}} \cdot \frac{1}{n} \end{aligned}$$

**Fig. 4:**
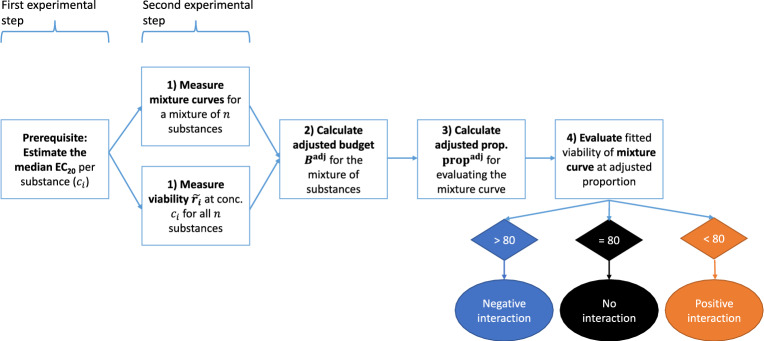
Flowchart summarizing the entire procedure of evaluating mixture curves concerning additivity, while adjusting for day-to-day variability in the estimation of effective concentrations, with the estimated reference median EC_20_ values per substance ($c_i$) as starting point, the different steps for the measuring of the mixture curves and the calculation of the adjusted proportions, and finally the evaluation of the fitted mixture curves with the resulting decision. The two-step experimental setup with the experiments corresponding to first and second step being conducted on different days, is clearly indicated

If $B^{\text {adj}} < 100\%$, the adjusted proportion is larger than $\frac{1}{n}$, and if $B^{\text {adj}} > 100\%$, then it holds $\text {prop}^{\text {adj}} < \frac{1}{n}$. The mixture curve is evaluated at this value and the response is compared to $80\%$. As introduced and explained above, a response value larger than $80\%$ at $\text {prop}^{\text {adj}}$ corresponds to a negative interaction effect, and a response value smaller than $80\%$ corresponds to a positive interaction effect. A complete summary of the procedure is shown in Fig. 4. An overview of the relevant notation for one substance only, i.e., with omitting the index *i*, is given as a Supplement.

### Sources of variation

The entire procedure of evaluating mixture curves concerning additivity consists of the determination of substance-wise reference median EC_20_ values $c_i$ from the reference curves (first experimental step), and the four steps determination of the day-specific viability ${\tilde{r}}_i$ at the concentration $c_i$ and measuring the mixture curves (second experimental step, performed on a different day than the measuring of the reference curves), the adjustment of the budget via the calculation of a value ${\tilde{c}}_i$, the adjustment of the proportion based on the adjusted budget, and finally the evaluation of the response of the mixture curve at the adjusted proportion (Fig. 4).

Above, in all these steps, only point estimates are considered, without taking any statistical variability into account. While rigorously deriving the extent of variability in the final analysis is not easily possible in a straightforward way and would exceed the scope of this work, this section aims to give an impression of the sources of variability and their influence on the final assessment of a potential interaction effect of the substances.

In the reference curves, the estimation of the EC_20_ value per curve has a variance. For a 4pLL model, the confidence interval of the resulting estimate can be approximated via the delta method (Jiang 2013; van der Vaart 1998), and it can be indicated in the respective plot. For the BC model, this is not possible in a straightforward way. For the determination of the reference EC_20_ value $c_i$, i.e., the median of all estimated EC_20_ values for one substance *i*, the variability between the individual experiments and the variability within the experiments (i.e., the confidence interval of the estimation of the EC_20_) is not taken into account. Instead, the reference EC_20_ value $c_i$ is further only considered to be the median of the point estimates.

For the assessment of day-to-day variability between the experiments, the response value ${\tilde{r}}_i$ is also taken as a median or mean of several technical replicates. Again, no statistical variability of these point estimates is considered.

When determining the concentration ${\tilde{c}}_i$ based on the estimated ${\tilde{r}}_i$ from the reference curve, again, the variability of the curve (on the y-axis) is not considered. The same is the case in the last step when the response value of the mixture curve is evaluated at the adjusted proportion.

Thus, overall, many sources of variation have an impact on the reliability of the estimate but are neglected during the procedure. To still take the sources of variation into account, we propose to not compare the response value of the fitted mixture curve at the adjusted proportion to a value of exactly $80\%$, but instead to use an interval of $[75\%, 85\%]$, within which it can be assumed that the combination of the substances indeed acts in an approximately additive way. This is not a formally derived interval of equivalence, but instead a reasonable application-relevant approximation.

## Application

To illustrate the proposed procedure with a specific dataset, a mixture of 10 substances is evaluated following the steps proposed above. Increasing proportions of the mixture and single concentrations (estimated reference EC_20_ values $c_i$) of the substances were applied to HepG2 cells and viability was determined using the CTB assay as described before. A detailed protocol for the CTB assay and information about the substances are provided in the Supplement.

The statistical analysis is conducted with the statistical software R, version 4.3.2 (R Core Team 2023) and the additional software package drc, version 3.0-1 (Ritz et al 2015), for fitting concentration–response and mixture curves.

The ten substances are acetaminophen (APAP), clonidine (CLON), cyclophosphamide (CYP), isoniazid (INAH), labetalol (LAB), levofloxacin (LEV), oxycodone (OXC), sodium phenylbutyrate (SPB), vitamin C (VITC), and valproic acid (VPA). Some of the original concentration–response curves were published before (Albrecht et al 2019), and the complete data are summarized in the Supplement. The resulting reference median EC_20_ values $c_i$ are summarized in Table 2. The reference experiments were conducted in three to six biological replicates with three technical replicates each, and five (in one case nine) concentrations plus a negative control. Details can be found in the Supplement in which all data are given.

**Table 2:** Summary of the ten considered substances in the mixture set, together with their respective reference median EC_20_ value $c_i$, and replicate-wise response values ${\tilde{r}}_i$ at the value $c_i$ alongside the respective concentration ${\tilde{c}}_i$, at which the response value ${\tilde{r}}_i$ is attained

	substance	APAP	CLON	CYP	INAH	LAB	LEV	OXC	SPB	VITC	VPA
	$c_i$ [mM]	3.3	0.5	3.4	8.9	0.035	0.4	0.6	8.3	1.5	1.4
Rep1	$\tilde{r_i}$ [%]	52.09	56.94	92.97	72.22	70.59	66.65	64.27	65.03	77.19	93.98
	$\tilde{c_i}$, Resp. [mM]	9.44	0.75	1.78	11.51	0.06	0.52	0.76	10.60	1.60	0.24
	$\tilde{c_i}$, Median [mM]	9.44	0.76	1.78	11.51	0.06	0.52	0.76	10.60	1.60	0.24
Rep2	$\tilde{r_i}$ [%]	62.47	63.55	77.66	63.00	72.10	73.04	61.45	34.65	83.63	71.08
	$\tilde{c_i}$, Resp. [mM]	6.88	0.67	3.62	15.17	0.05	0.45	0.79	15.94	1.36	2.44
	$\tilde{c_i}$, Median [mM]	6.88	0.69	3.62	15.17	0.05	0.45	0.79	15.36	1.36	2.44
Rep3	$\tilde{r_i}$ [%]	66.43	72.49	83.46	57.38	85.39	61.35	75.63	42.45	87.34	79.29
	$\tilde{c_i}$, Resp. [mM]	5.99	0.57	2.97	17.84	0.02	0.59	0.64	14.31	1.21	1.44
	$\tilde{c_i}$, Median [mM]	6.04	0.57	2.97	17.84	0.03	0.59	0.64	14.03	1.21	1.44

The mixture experiments are conducted in three independent biological replicates, for a negative control (i.e., a mixture proportion of 0), and eight increasing mixture proportions, with four technical replicates each. Following the typical notation, the exact design $\xi$ with $N=36$ samples overall of one biological replicate of the mixture experiments is defined as

$$\xi = \begin{pmatrix} 0 & \nicefrac {1}{128} & \nicefrac {1}{64} & \nicefrac {1}{32} & \nicefrac {1}{16} & \nicefrac {1}{8} & \nicefrac {1}{4} & \nicefrac {1}{2} & 1 \\ \nicefrac {4}{36} & \nicefrac {4}{36} & \nicefrac {4}{36} & \nicefrac {4}{36} & \nicefrac {4}{36} & \nicefrac {4}{36} & \nicefrac {4}{36} & \nicefrac {4}{36} & \nicefrac {4}{36} \end{pmatrix}.$$

Here, the support points (first row) in the design correspond to the mixture proportions, i.e., the administered concentrations are calculated as the respective weight times the reference EC_20_-based mixture of all substance, e.g., as $\frac{1}{128}\cdot \sum _{i=1}^{10}{c_i}$ for the lowest proportion. The highest tested mixture proportion is combining the reference EC_20_ values $c_i$ for all 10 substances, i.e., $1 \cdot \sum _{i=1}^{10}{c_i}$. The weights (second row) indicate that, for the considered $N=36$ samples, an equal number of 4 technical replicates are measured per mixture proportion.

The corresponding experiments for the individual substances $i=1, \ldots , 10$ for the day-specific viabilities ${\tilde{r}}_i$ measured at the concentrations $c_i$ are conducted in the same three biological replicates as the mixture experiments, with two technical replicates each, and a negative control measured in four technical replicates. In the typical notation, for substance *i*, this corresponds to an exact design $\xi _i$ with $N=6$ samples for each biological replicate with

$$\xi _i = \begin{pmatrix} 0 & c_i \\ \nicefrac {4}{6} & \nicefrac {2}{6} \end{pmatrix}, \quad i=1, \ldots , 10.$$

**Fig. 5:**
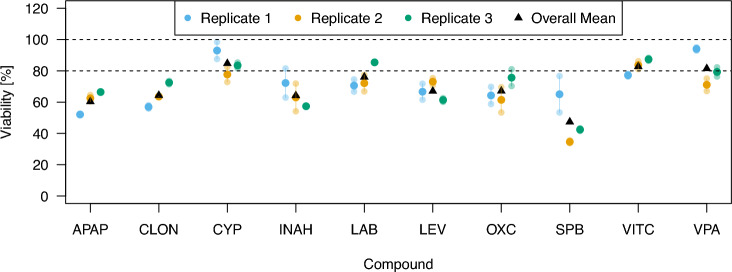
The day-specific viabilities ${\tilde{r}}_i$ are shown for all ten substances in the considered mixture set. The two technical replicates per biological replicate and their mean value are displayed alongside the overall mean of all six replicates per substance

As a first step, the day-specific viabilities ${\tilde{r}}_i$ at the concentrations $c_i$ are normalized with respect to the mean of four technical replicates of experiment-wise controls. The substance-wise ${\tilde{r}}_i$ is calculated as the mean value of the two technical replicates, which in this case coincides with the median. The response values are shown in Fig. 5. Notably, several substances lead to far lower viability values than the expected $80\%$, and only three substances have viabilities slightly above $80\%$. This indicates that for the majority of substances, an individual budget of more than 100% is spent, and thus within a mixture based on the individual reference EC_20_ values $c_i$, overall more than $100\%$ of the intended toxicity budget was spent.

The second step is the calculation of the adjusted budget. For this the determination of the values ${\tilde{c}}_i$ based on the reference curves is necessary, i.e., determining the concentration at which the reference curve intersects with the previously calculated response value ${\tilde{r}}_i$. Two different approaches were proposed above: Either, only the curve responsible for the median EC_20_ value is considered (**Responsible**), or all curves are considered and the median is taken afterwards (**Median**). The different values for ${\tilde{r}}_i$ as shown in Fig. 5 are also summarized in Table 2.

**Fig. 6:**
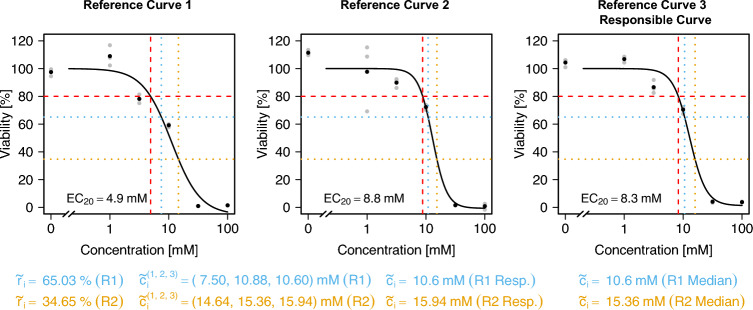
Example of the three reference concentration–response curves for the substance SPB, together with the respective estimates of the EC_20_ (red dashed line). The third curve is the curve responsible for the median value of the EC_20_. For the day-specific viabilities of replicates 1 (light blue) and 2 (orange), the determination of the values ${\tilde{c}}_i^{(j)}$ are displayed. Finally, this leads to the determination of the value ${\tilde{c}}_i$ for the substance SPB for the **Responsible** and the **Median** approach

Calculation of the values ${\tilde{c}}_i$ based on the reference curves only for the first and second replicate of the independent experiments is explicitly displayed for the substance SPB in Fig. 6. The third replicate is not explicitly displayed here, but the procedure works analogously. SPB was measured with three concentration–response curves in the reference experiments. The respective values of the resulting EC_20_ values are indicated in the plot. Here, the third reference curve was responsible for the calculation of the reference median EC_20_ value $c_i$, since the median of the three values 4.9, 8.8 and 8.3 is given by 8.3. For the first replicate (Rep1) of the experiment to determine ${\tilde{r}}_i$ on the same day as the mixture experiment is conducted, the day-specific viability at concentration $c_i$ corresponds to $65.03\%$, and to $34.65\%$ for the second replicate (Rep2, see Fig. 5 and Table 2). When considering the responsible curve only, i.e., the third reference curve, these response values correspond to concentrations of 10.60 mM (Rep1) and 15.94 mM (Rep2). When using the median approach, the concentrations are 10.60 mM (Rep1) and 15.36 mM (Rep2). Thus, when considering Rep1, the value for ${\tilde{c}}_i$ does not depend on the approach (**Responsible** vs. **Median**), while for Rep2 the values for ${\tilde{c}}_i$ differ slightly. For the third replicate, which is not shown here, the two approaches also lead to different values of ${\tilde{c}}_i$ (see Table 2).

The resulting values of ${\tilde{c}}_i$ for all substances in the different approaches are summarized in Table 2. Generally, it can be observed that the approaches **Responsible** and **Median** often lead to the same results for ${\tilde{c}}_i$ and if they differ, then only by a small amount. Comparing the values for ${\tilde{c}}_i$ to the reference EC_20_ values $c_i$ (Table 2), an increase in ${\tilde{c}}_i$ in comparison to $c_i$ can be seen for those substances that have higher day-specific cytotoxicity (i.e., ${\tilde{r}}_i < 80\%$), and vice versa. This reflects the basic idea of the approach, where we assume the reference curve to represent the true concentration–response relationship, and thus the budget is adjusted based on this curve according to the day-specific viability.

Given the different values ${\tilde{c}}_i$, the substance-wise adjusted budgets $B_i^{\text {adj}}$ are calculated according to Equation (1). These values $B_i^{\text {adj}}$ are summarized in Table 3 for both approaches. The values for ${\tilde{c}}_i$ often coincide for the approaches **Responsible** and **Median**, and in such cases also the respective values for $B_i^{\text {adj}}$ are the same.

**Table 3:** Substance- and replicate-wise adjusted budget (rounded to integers) for the 10 substances in the mixture, for both approaches **Responsible** and **Median**

	substance	APAP	CLON	CYP	INAH	LAB	LEV	OXC	SPB	VITC	VPA
Rep1	$B_i^{\text {adj}}$, Resp	286	150	52	129	163	131	127	128	107	17
	$B_i^{\text {adj}}$, Median	286	152	52	129	163	131	127	128	107	17
Rep2	$B_i^{\text {adj}}$, Resp	208	134	106	170	152	112	132	192	91	174
	$B_i^{\text {adj}}$, Median	208	137	106	170	152	112	132	185	91	174
Rep3	$B_i^{\text {adj}}$, Resp	182	114	87	200	67	147	106	172	81	103
	$B_i^{\text {adj}}$, Median	183	114	87	200	78	147	106	169	81	103

The overall adjusted budget is then calculated as the mean of the component-wise adjusted budgets. Since $n=10$ substances are considered in the mixture, without day-to-day variability, the mixture curves would have to be evaluated at a proportion of 0.1. This proportion is adjusted according to Equation (3). The overall adjusted budgets and proportions are summarized in Table 4.

**Table 4:** Overall adjusted budget administered to cells in the mixture experiment for the three replicates and the two approaches **Responsible** and **Median**, together with the corresponding adjusted proportions. The last column states the response values, i.e., the viabilities, for the mixture curves evaluated at the respective adjusted proportions

	Approach	$B^{\text {adj}}$	$\text {prop}^{\text {adj}}$	Viability [%]
Rep1	Responsible	128.922	0.078	95.9
	Median	129.105	0.077	95.9
Rep2	Responsible	147.257	0.068	87.4
	Median	146.908	0.068	87.3
Rep3	Responsible	125.971	0.079	85.4
	Median	126.847	0.079	85.6

**Fig. 7:**
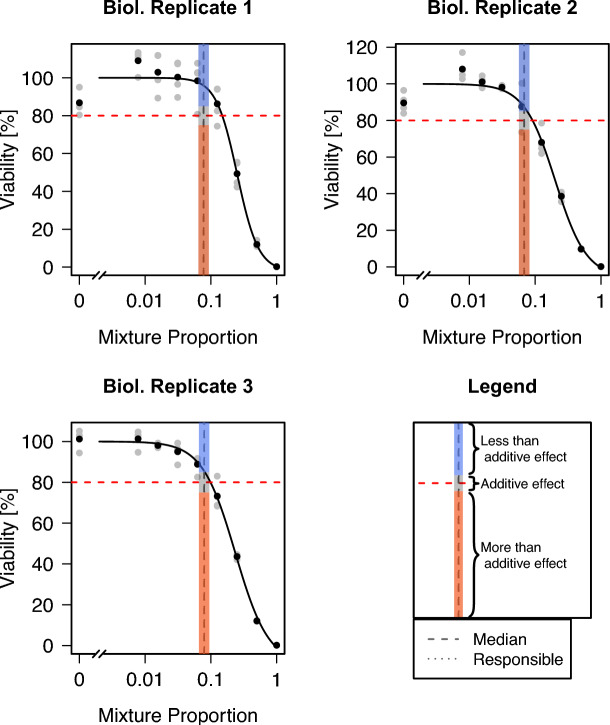
All three biological replicates of mixture curves show a negative (less than additive) interaction effect, because the fitted mixture curves, evaluated at the adjusted proportions, show a predicted viability above 85%. The mixtures consist of a set of 10 substances. The red dashed line indicates the response value of $80\%$. The dashed and dotted lines show the adjusted proportion for the two approaches **Median** and **Responsible**. Since both approaches lead to similar results, these lines overlay each other. The response values at the adjusted proportion are colored according to the interpretation of the results. If the fitted mixture curve at the adjusted proportion corresponds to a viability larger than 85% (blue area), a less than additive effect is present, if it corresponds to a viability between 75% and 85% (black area), an additive effect is present, and for viabilities below 75% (red area), a more than additive effect is present

The mixture curves are fitted as described above and evaluated at the adjusted proportions $\text {prop}^{\text {adj}}$ indicated in Table 4. The resulting models are shown in Fig. 7. Here, the adjusted proportions at which the response values of the fitted curve must be evaluated are also shown. The respective predicted viabilities at the individual adjusted proportions for the three replicates and both approaches are summarized in Table 4.

Especially for the first replicate, the predicted viabilities are far higher than $80\%$, indicating a negative interaction effect. For the second and third replicates, the predicted viabilities are lower, but still, all take at least a value of $85\%$, also indicating negative interactions.

For illustrative purposes, this procedure and all its intermediate steps are presented here in much more detail than would be needed to interpret the results. For interpretation of the final results, only the fitted curves and their predicted viability values at the adjusted proportions are of main interest to decide whether an interaction effect is present.

Overall, in this specific set of 10 substances, the interaction of the substances seems to be negative, i.e., they have a less than additive effect. Evaluated at the adjusted proportion, at which under the assumption of additivity in principle a predicted viability of approximately $80\%$ would be expected, a higher viability of above $85\%$ or even above $95\%$ (Replicate 1) is observed.

## Discussion

When assessing the toxicity of mixtures, data of the individual substances and of combined exposures are required. This leads to the necessity of testing high numbers of conditions, which cannot be accomplished on a single experimental day. For example, testing 100 individual substances at seven concentrations and three technical replicates results in 2,100 conditions, each requiring a ‘well’ during the practical implementation of the test. This number increases massively if additionally combinations of the substances are tested. For testing of high numbers of conditions, typically 96- or 384-well dishes are used in laboratory routine, and the number of such dishes that can be tested on a single day is limited. Even with the support of pipetting machines, it may be impossible to complete the tests on a single experimental day. This leads to the challenge to control for day-to-day variability which typically is relatively high in cytotoxicity testing, certainly much higher than the variability of several technical replicates of a single experiment. In daily routine, it is often observed that single days or even some consecutive days result in higher or lower day-specific toxicities (measured, e.g., by the EC_20_) compared to the data obtained some weeks later. This can result in the unfavorable constellation that the individual substances are tested at experimental days with a different level of toxicity compared to the experimental days when the mixture is tested. This problem is of practical importance because it is the only feasible way to use the toxicity data of the individual substances from historical experiments while the mixtures are tested independently later. Since it would result in an unmanageable number of conditions if all concentrations of the individual substances would have to be tested on the same experimental day as the mixtures, a possible solution is to include (at least) one well-chosen single concentration of each substance at the experimental day when a mixture is tested. In this work, the experiment is repeated at the previously determined alert concentration EC_20_, which is also used as basis of the mixture experiments. This approach substantially reduces the number of required conditions so that testing on a single day becomes possible. We proposed an approach for including such day-to-day controls and conclude that ignoring the day-to-day variability may lead to a massive over- or underestimation of mixture effects.

To infer about positive or negative interaction effects for a mixture of substances, an additive reference model is required. In this paper, an in vitro-based approach to determine the mixture toxicity of a set of substances is proposed. This approach is based on the measurement of an entire proportion–mixture curve, where the predictor is a proportion of individual EC_20_ values of the substances in the set, and the response is given by the viabilities of cells. Under the assumption of additivity, such a mixture curve evaluated at the proportion of $\frac{1}{n}$, where *n* denotes the number of substances in the mixture set, should yield a response value of $80\%$. We denote this as the *budget approach*. It fits into the established additivity model as introduced by Loewe (1927), but is extended to a higher number of substances.

A similar approach has, e.g., been followed by Backhaus et al (2000) and Faust et al (2001), who tested 10 and 18 compounds, respectively, relevant in the context of aquaculture, based on individual concentration–response curves and mixture curves for mixture toxicity. Effective concentrations (EC values) and no observed effect concentrations were considered as the basis for the mixtures, and mixtures were analyzed with respect to the reference model by Loewe additivity and Bliss independence. In both cases, the authors came to the conclusion that the Loewe additivity model is very suitable for predicting the mixture toxicity for both sets of compounds. Here, the sets of compounds are very homogeneous by acting as inhibitors of the same processes, respectively.

A set of only four compounds, however more similar to the compounds tested in the case study presented in this paper, was considered by Cleuvers (2004). The four compounds are considered to have the same mode of action. Also here, Loewe additivity is used as a framework and concluded to be very suitable for the specific application.

More recently, Kim et al (2024) advocated for conducting many experiments with mixtures of several instead of only two or three substances. These ‘complex mixtures’ might contain substances acting via different modes of action. In the work, the authors analyzed 25 single substances with concentration–response curves and randomly generated 50 mixtures with 7 to 10 substances each, which were evaluated based on mixture–response curves as well. The data were analyzed based on both Loewe additivity and Bliss independence, however the Loewe additivity approach was shown to be more accurate for mixture toxicity prediction.

In all these publications, mainly additive effects were observed, fitting to the results from the review by Cedergreen (2014). Additionally, all the publications have in common that they do not take variability between the independent experiments into account.

In real data, the phenomenon of day-to-day variability is observed due to the special experimental setup required for analyzing mixtures of many substances. To assess the toxicity of a mixture, substance-wise reference toxicity markers (here: EC_20_ values) are calculated based on an initial set of at least 3 independent experiments. The substance-wise reference EC_20_ values are combined in fixed proportions to obtain mixtures, and a parametric curve can be fitted to the relationship between proportion and resulting viability. The mixture experiments are typically conducted on different days in comparison to the reference experiments, leading to some day-to-day variability in the observed viability. Thus, a higher or lower cytotoxicity may be induced in the experiment where the mixture is tested compared to the previous reference experiments which is typically not accounted for in the literature. It is however important to note that the data from the reference curves are not pooled or analyzed together with the data of the mixture curves. The reference curves are only used for the determination of the EC_20_ values, which then serve as a basis for the mixture curves. Based on additional individual measurements of the substance-wise reference EC_20_ values within the same experiment as the mixture, we propose an adjustment approach to take the day-to-day variability into account when analyzing mixture curves.

To illustrate the procedure in detail, a case study with a mixture consisting of 10 substances is presented. For almost all substances, day-specific cytotoxicity was observed to be slightly higher in comparison to the reference experiments. Thus, the entire mixture curve is analyzed with adjustments for this day-to-day variability. For all three biological replicates of the mixture experiments, this ultimately resulted in the conclusion of a negative interaction effect, i.e., the combination of substances at an adjusted proportion was less cytotoxic than each substance at its individual EC_20_.

In the context of statistical design theory, the design chosen in the here presented experiment is a special case of a ray design with equal mixture proportion and log-equidistant x-axis values (Holland-Letz and Kopp-Schneider 2021). Generally, within a ray design, a fixed proportion of certain dose levels, in the case presented here of the EC_20_ value, for several substances is chosen, and curves for increasing overall doses are measured. Holland-Letz and Kopp-Schneider (2021) derive an optimal design in terms of the proportion of the dose levels at which the viability should be measured within a pre-specified ray, i.e., a fixed mixture proportion, in the context of Loewe additivity (Loewe 1927), for two substances. However, these designs cannot be used to investigate the response of different mixture proportions. In particular, thresholds between toxic and non-toxic combinations of the two substances cannot be determined. Here, further research is needed to define appropriate design criteria that address this question.

In this work, the mixture is based on a combination of absolute EC_20_ values of the individual substances, since this value is more robust in comparison, e.g., to the EC_10_, and fewer problems in solubility especially for higher mixture proportions are expected in comparison, e.g., to the commonly used EC_50_. The choice of absolute EC values (i.e., the concentration where a fixed viability percentage is attained) in comparison to relative values (i.e., the concentration where some percentage of the overall effect is attained) is motivated by the fact that the lower asymptotes of viability curves often do no exactly correspond to zero. In these cases, a direct interpretation of relative EC values is not possible. However, an extension to any other effective concentration or other parameters, i.e., any other alert concentration, derived from the original concentration–response curves is straightforward.

## Supplementary information

Additional supplementary information, containing SOPs and raw data, are available online.

## Supplementary Information

Below is the link to the electronic supplementary material.

Supplementary file 1 (pdf 90 KB)

Supplementary file 2 (pdf 148 KB)

Supplementary file 3 (pdf 83 KB)

Supplementary file 4 (xlsx 30 KB)

Supplementary file 5 (docx 430 KB)

## Data Availability

Raw data are made available in the Supplement (available online). Pre-processed data are uploaded alongside the code in a github repository (see below).

## Code availability

The code for fitting the reference and mixture curves and adjusting for day-to-day variability, alongside all pre-processed data, is uploaded in the github repository https://github.com/FKappenberg/MixtureCytotox-DayToDayVariabilityAdjustment
