# Supplementary material for: Adjustment for day-to-day variability in the estimation of effective concentrations for the assessment of mixture toxicity
Source: Arch Toxicol. 2025 Aug 19;99(11):4439–54. doi: 10.1007/s00204-025-04141-w (PMC12477094; doi:10.1007/s00204-025-04141-w)
Supplement: Supplementary file 1 — (pdf 90 KB) [file 204_2025_4141_MOESM1_ESM.pdf]

Supporting Information for ‘Adjustment for day-to-day variability in the estimation of effective concentrations for the assessment of mixture toxicity’

Additional formulas

Within the fitting of the concentration-response curves, flat model, the sigmoidal and monotonous four-parametric-log-logistic (4pLL) (Ritz et al., 2019), and the non-monotonous Brain-Cousens (BC) (Brain and Cousens, 1989) model are fitted to the pre-processed data.

The formulas for the 4pLL and the BC models are as follows:

$$f_{\text{4pLL}}(x) = c + \frac{d - c}{1 + \exp(b \cdot (\log(x) - \log(e))} \tag{1}$$

$$f_{\text{BC}}(x) = c + \frac{d - c + f \cdot x}{1 + \exp(b \cdot (\log(x) - \log(e))} \tag{2}$$

For both models, c and d are the lower and upper asymptote, respectively. For the BC model, the parameters b and e do not have a direct interpretation, and the parameter f denotes the strength of the hormesis effect, i.e., the increase of the concentration-response curve before the decrease. For the 4pLL model, the parameter e denotes the half-maximal effect concentration, which is at the same time the inflection point of the curve. Parameter b is the slope parameter and is proportional to the gradient of the curve at concentration e.

Figures A.1 and A.2 show some exemplary profiles of 4pLL and BC models with different parameter combinations.

References

Brain, P. and Cousens, R. D. (1989). An equation to describe dose responses where there is stimulation of growth at low dose. *Weed research*, 29:93–96.

Ritz, C., Jensen, S. M., Gerhard, D., and Streiberg, J. C. (2019). *Dose-Response Analysis Using R*. Chapman and Hall / CRC, New York.

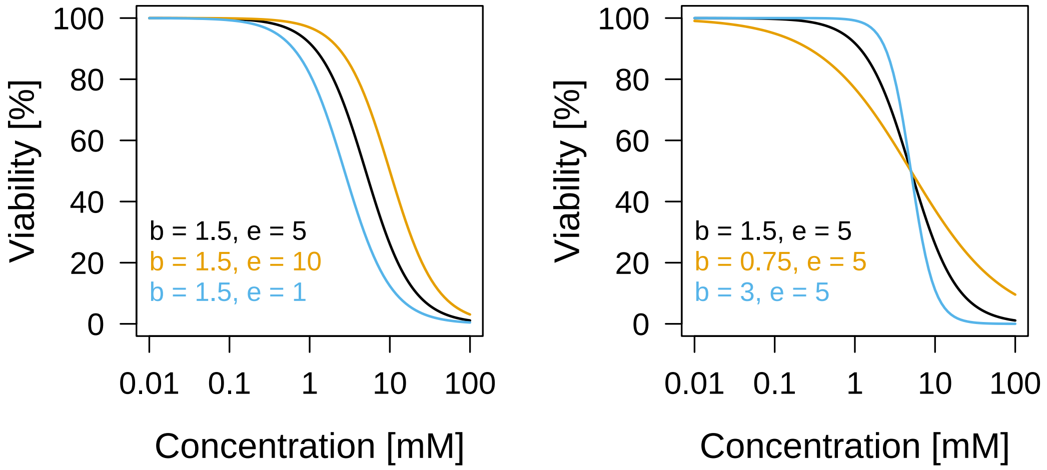

Figure A.1: Exemplary display of 4pLL models with different values for parameters b and e. The asymptote parameters c and d are fixed to take values of 0 and 100, respectively.

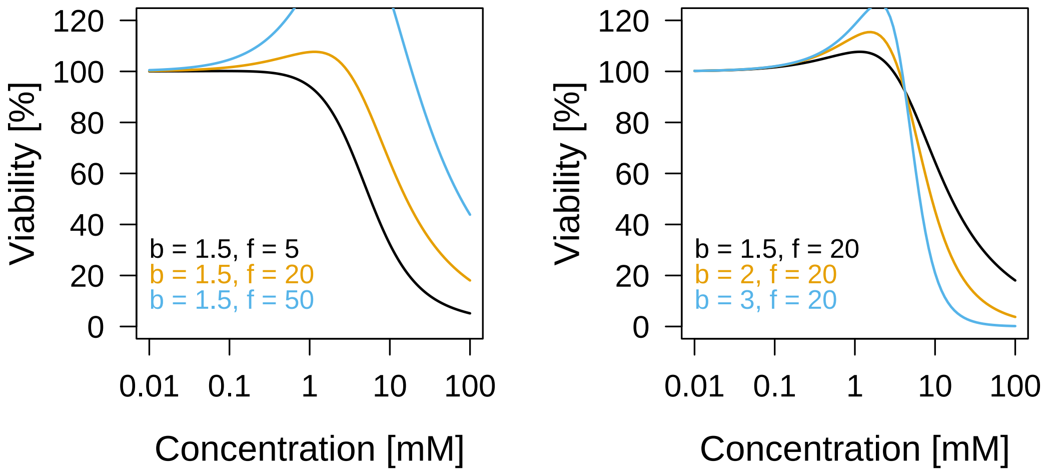

Figure A.2: Exemplary display of BC models with different values for parameters b and f. The asymptote parameters c and d are fixed to take values of 0 and 100, respectively, and parameter e is fixed to take a value of 4.
