# Supplementary material for: Adjustment for day-to-day variability in the estimation of effective concentrations for the assessment of mixture toxicity
Source: Arch Toxicol. 2025 Aug 19;99(11):4439–54. doi: 10.1007/s00204-025-04141-w (PMC12477094; doi:10.1007/s00204-025-04141-w)
Supplement: Supplementary file 2 — (pdf 148 KB) [file 204_2025_4141_MOESM2_ESM.pdf]

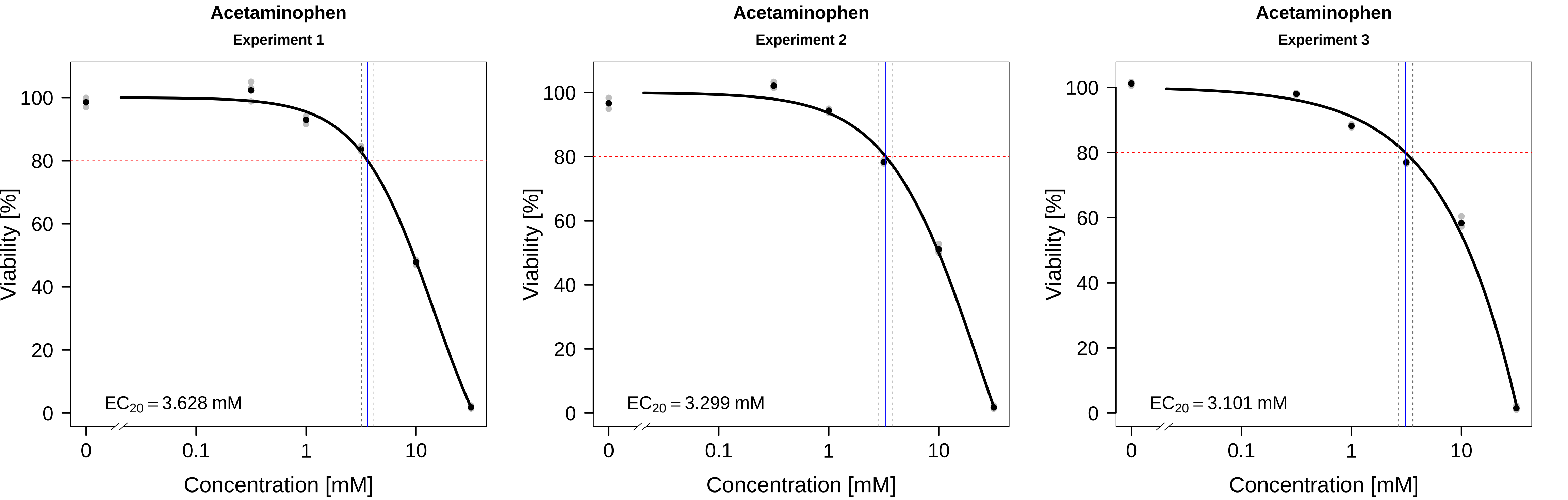

Acetaminophen
Experiment 1
$EC_{20} = 3.628$ mM
Acetaminophen
Experiment 2
$EC_{20} = 3.299$ mM
Acetaminophen
Experiment 3
$EC_{20} = 3.101$ mM
Viability [%]
Concentration [mM]
0
0.1
1
10
100
80
60
40
20

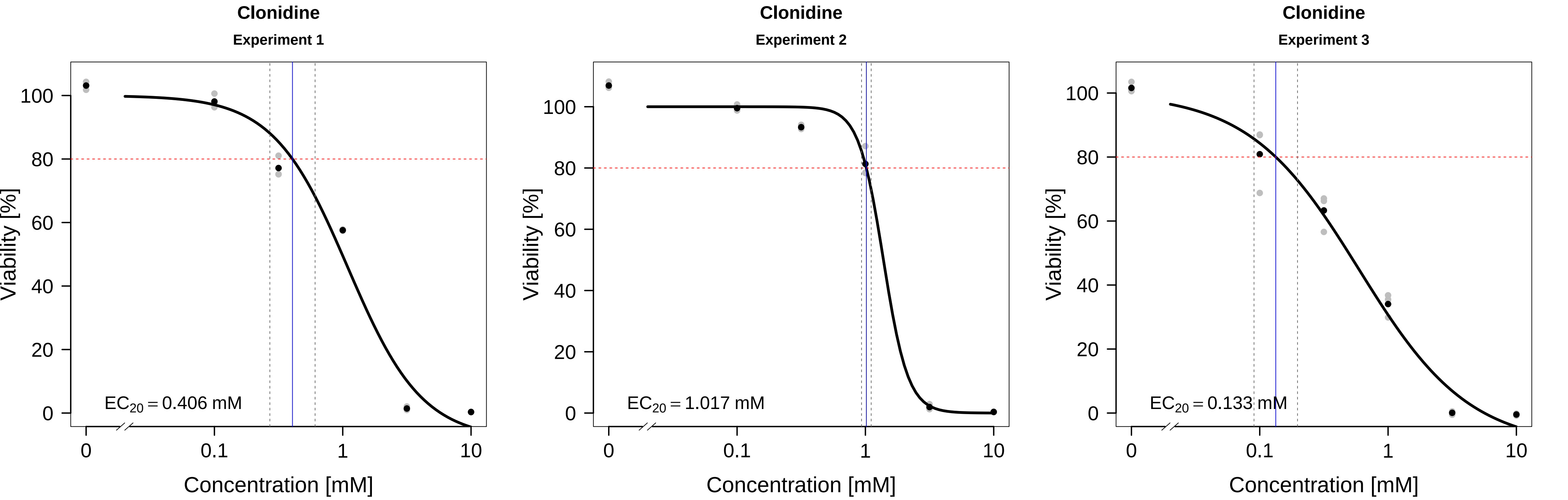

Clonidine
Experiment 1
Viability [%]
Concentration [mM]
$EC_{20} = 0.406$ mM
Clonidine
Experiment 2
Viability [%]
Concentration [mM]
$EC_{20} = 1.017$ mM
Clonidine
Experiment 3
Viability [%]
Concentration [mM]
$EC_{20} = 0.133$ mM

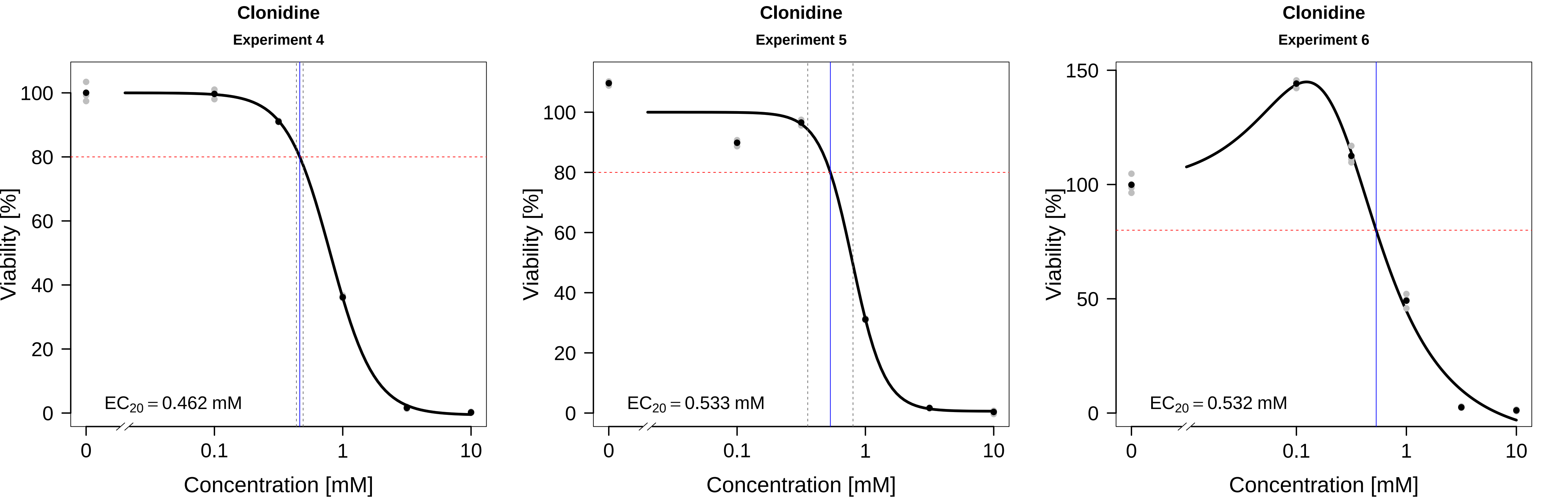
Clonidine
Experiment 4
Viability [%]
Concentration [mM]
EC20 = 0.462 mM
0
20
40
60
80
100
0
0.1
1
10
Clonidine
Experiment 5
Viability [%]
Concentration [mM]
EC20 = 0.533 mM
0
20
40
60
80
100
0
0.1
1
10
Clonidine
Experiment 6
Viability [%]
Concentration [mM]
EC20 = 0.532 mM
0
50
100
150
0
0.1
1
10

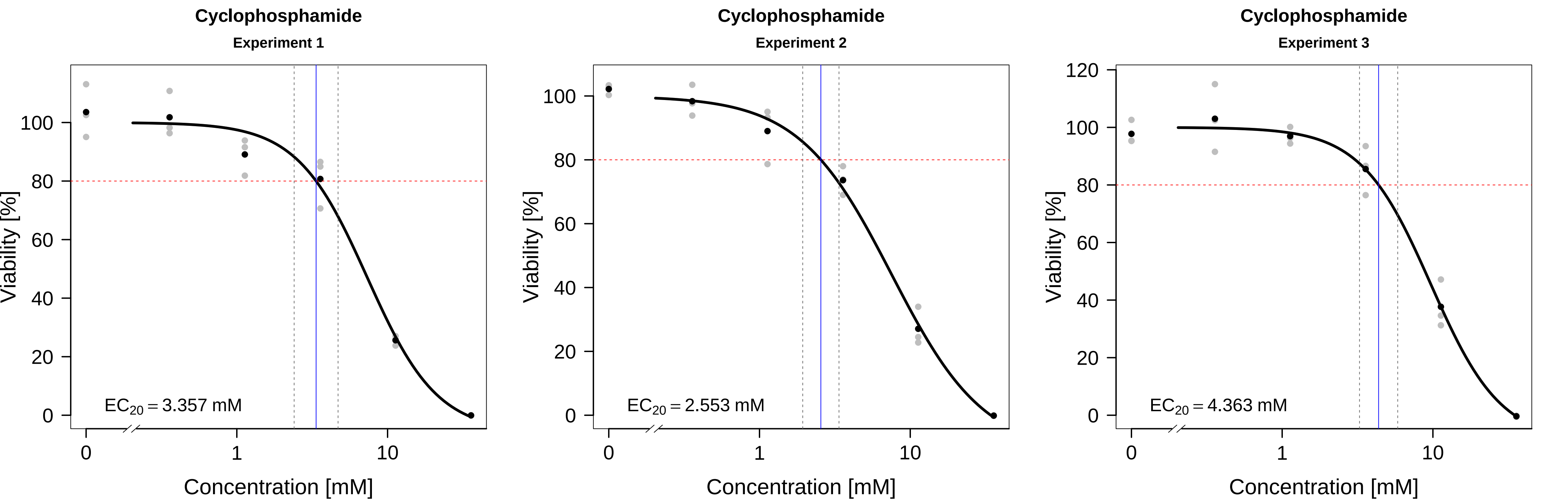

Cyclophosphamide
Experiment 1
$EC_{20} = 3.357$ mM
Cyclophosphamide
Experiment 2
$EC_{20} = 2.553$ mM
Cyclophosphamide
Experiment 3
$EC_{20} = 4.363$ mM
Viability [%]
Concentration [mM]
0
1
10

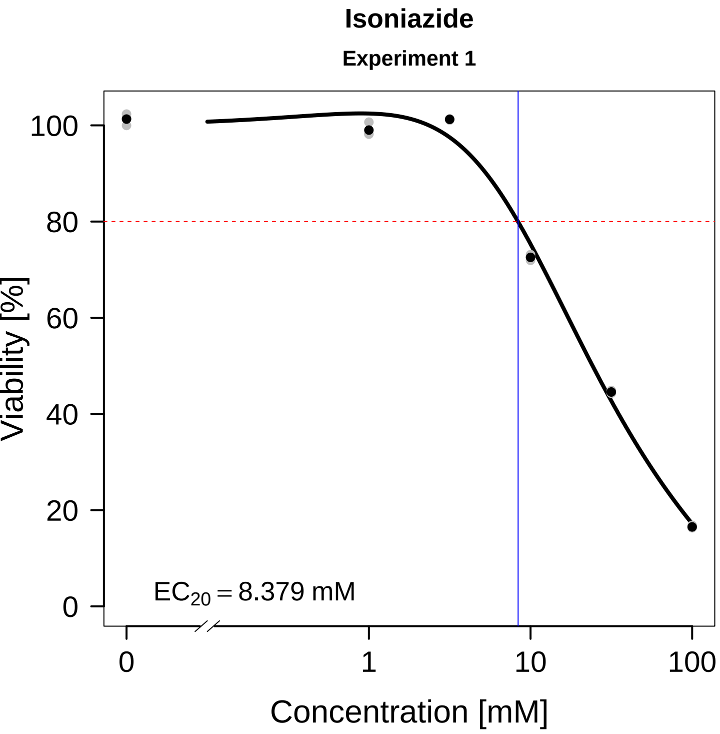
Isoniazide
Experiment 1
Viability [%]
100
80
60
40
20
0
$EC_{20} = 8.379$ mM
0
1
10
100
Concentration [mM]

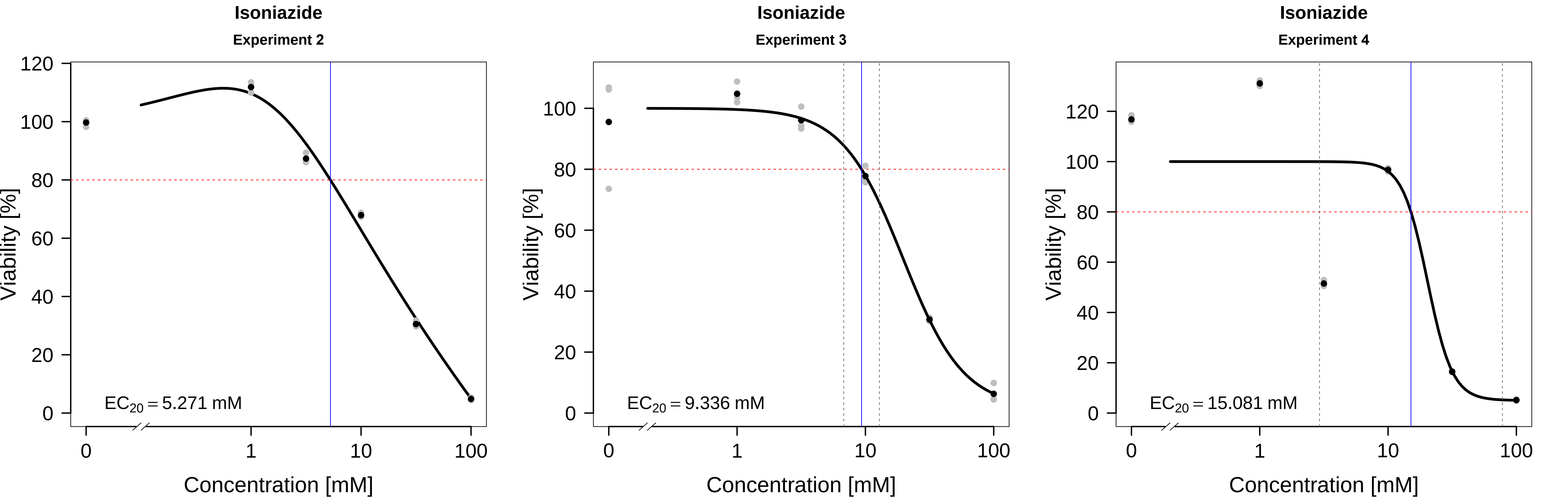
Isoniazide
Experiment 2
EC20 = 5.271 mM
Isoniazide
Experiment 3
EC20 = 9.336 mM
Isoniazide
Experiment 4
EC20 = 15.081 mM
Viability [%]
Concentration [mM]

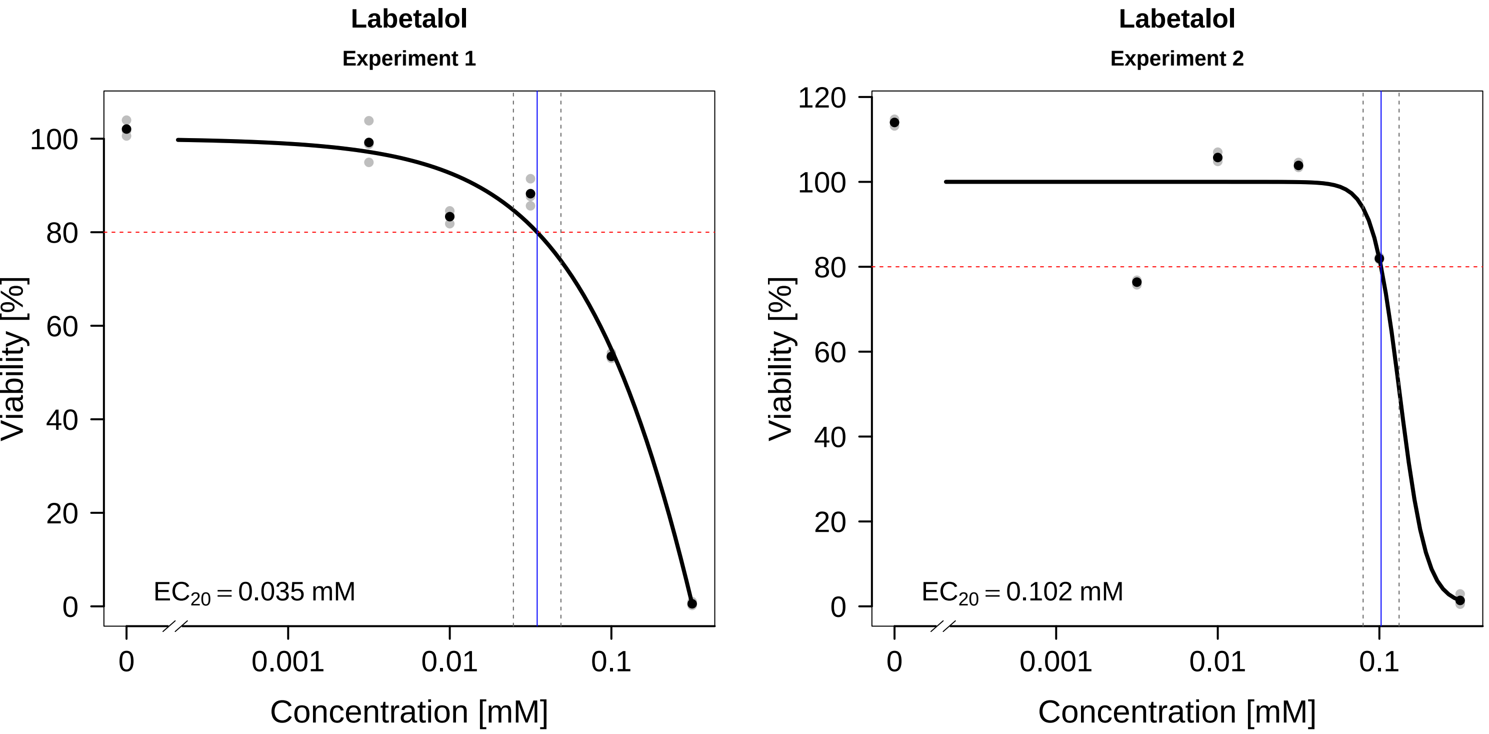

Labetalol
Experiment 1
Viability [%]
100
80
60
40
20
0
EC20 = 0.035 mM
0
0.001
0.01
0.1
Concentration [mM]
Labetalol
Experiment 2
Viability [%]
120
100
80
60
40
20
0
EC20 = 0.102 mM
0
0.001
0.01
0.1
Concentration [mM]

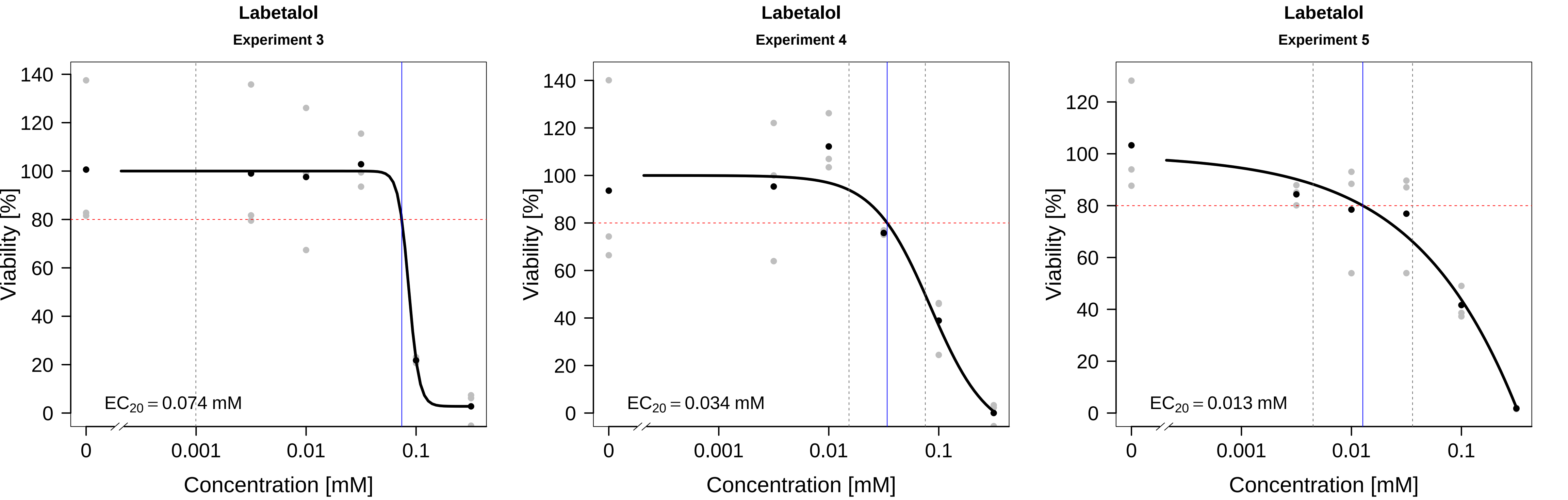

Labetalol
Experiment 3
Viability [%]
Concentration [mM]
EC20 = 0.074 mM
0
0.001
0.01
0.1
Labetalol
Experiment 4
Viability [%]
Concentration [mM]
EC20 = 0.034 mM
Labetalol
Experiment 5
Viability [%]
Concentration [mM]
EC20 = 0.013 mM

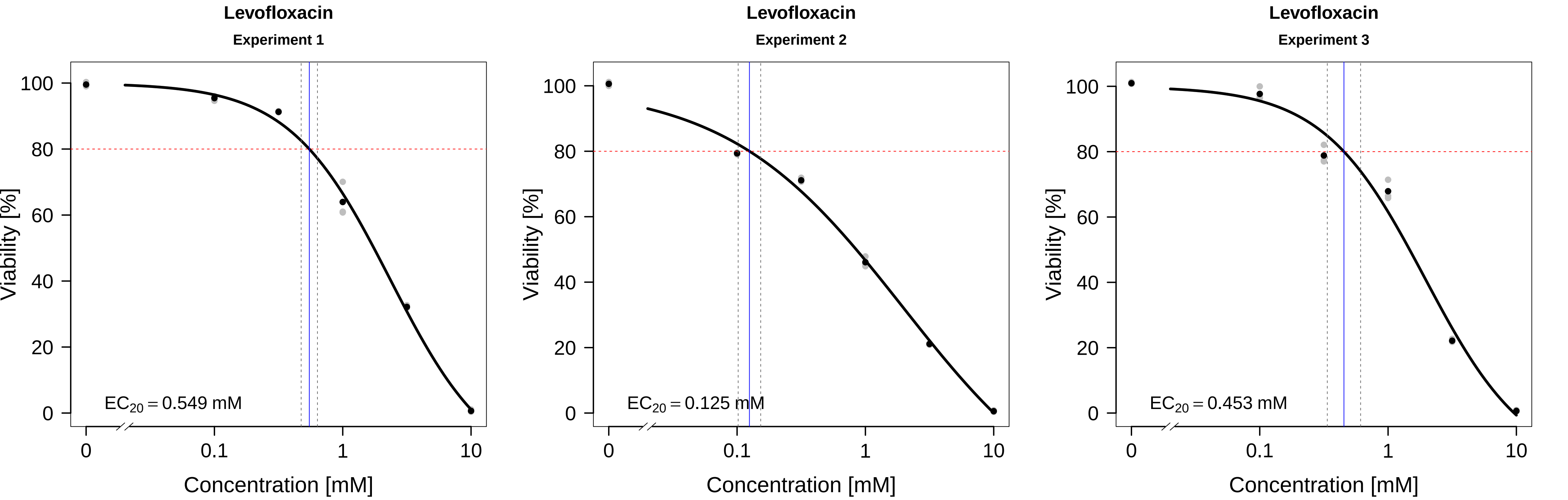

Levofloxacin
Experiment 1
Viability [%]
100
80
60
40
20
0
0
0.1
1
10
Concentration [mM]
EC20 = 0.549 mM
Levofloxacin
Experiment 2
Viability [%]
100
80
60
40
20
0
0
0.1
1
10
Concentration [mM]
EC20 = 0.125 mM
Levofloxacin
Experiment 3
Viability [%]
100
80
60
40
20
0
0
0.1
1
10
Concentration [mM]
EC20 = 0.453 mM

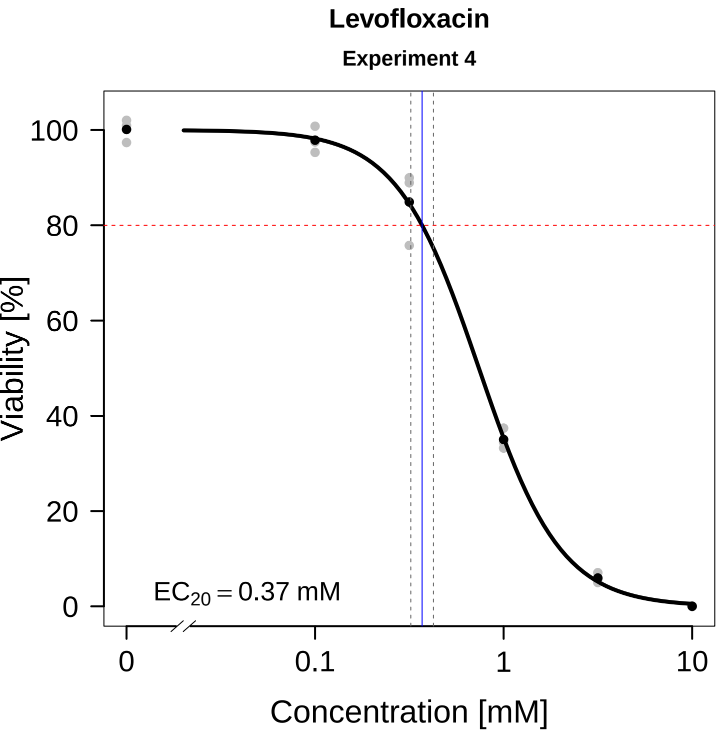
Levofloxacin
Experiment 4
Viability [%]
100
80
60
40
20
0
0
0.1
1
10
Concentration [mM]
$EC_{20} = 0.37$ mM

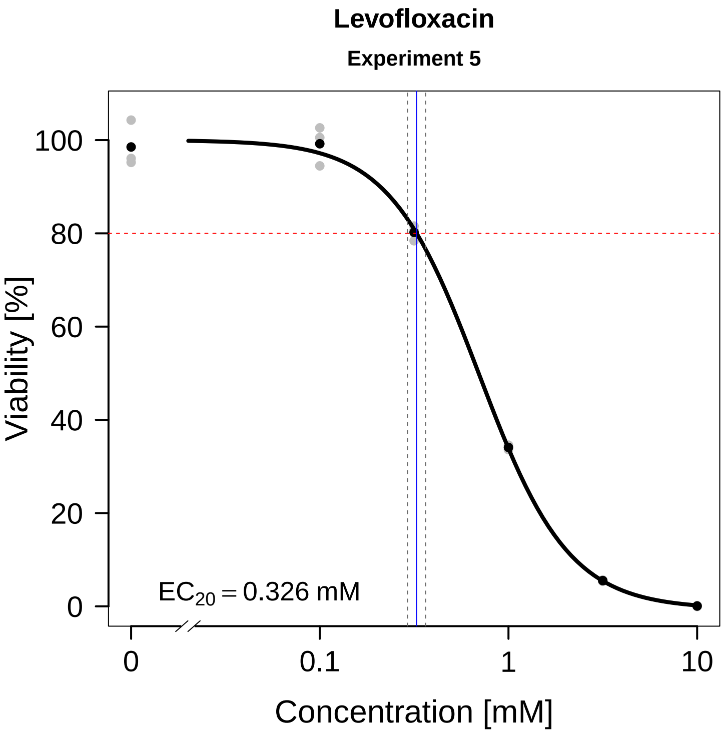
Levofloxacin
Experiment 5
Viability [%]
100
80
60
40
20
0
0
0.1
1
10
Concentration [mM]
$EC_{20} = 0.326$ mM

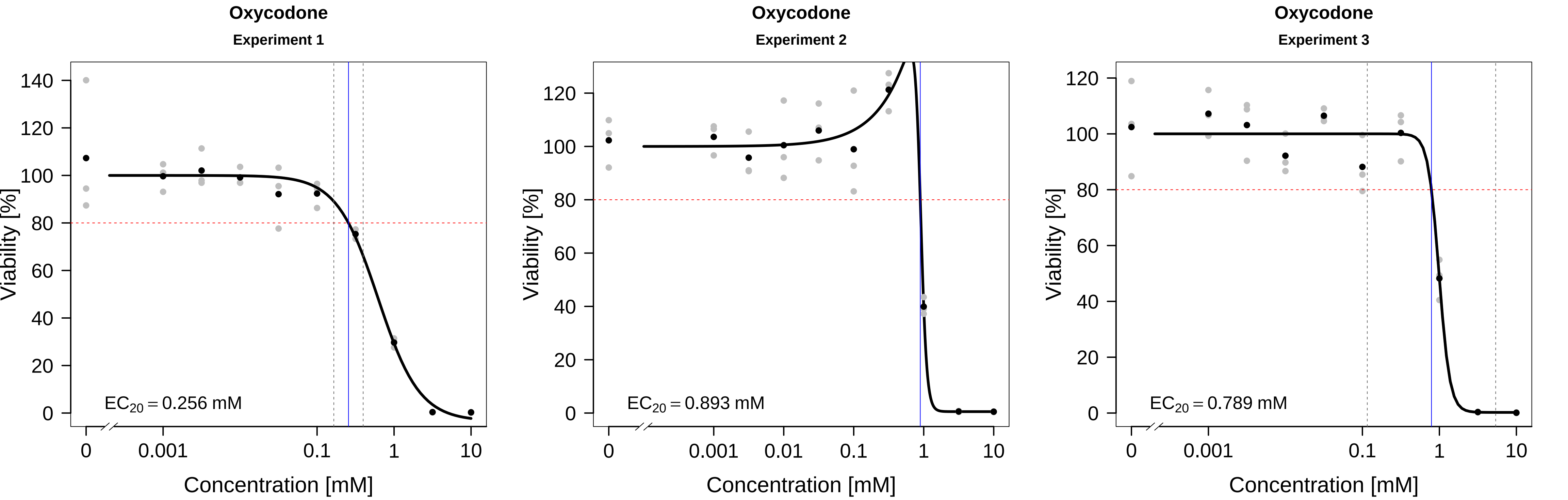

Oxycodone
Experiment 1
EC20 = 0.256 mM
Oxycodone
Experiment 2
EC20 = 0.893 mM
Oxycodone
Experiment 3
EC20 = 0.789 mM
Viability [%]
Concentration [mM]
0
0.001
0.01
0.1
1
10

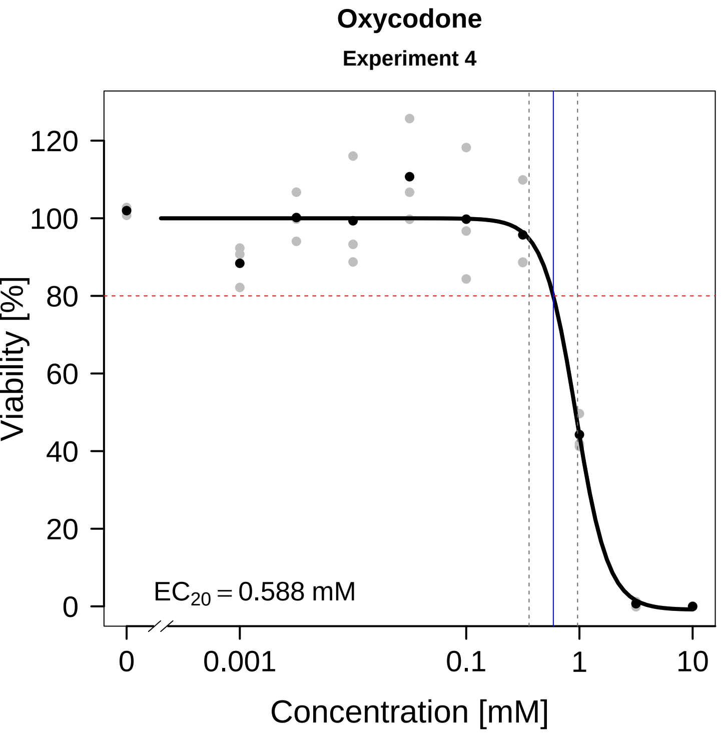
Oxycodone
Experiment 4
Viability [%]
120
100
80
60
40
20
0
EC20 = 0.588 mM
0
0.001
0.1
1
10
Concentration [mM]

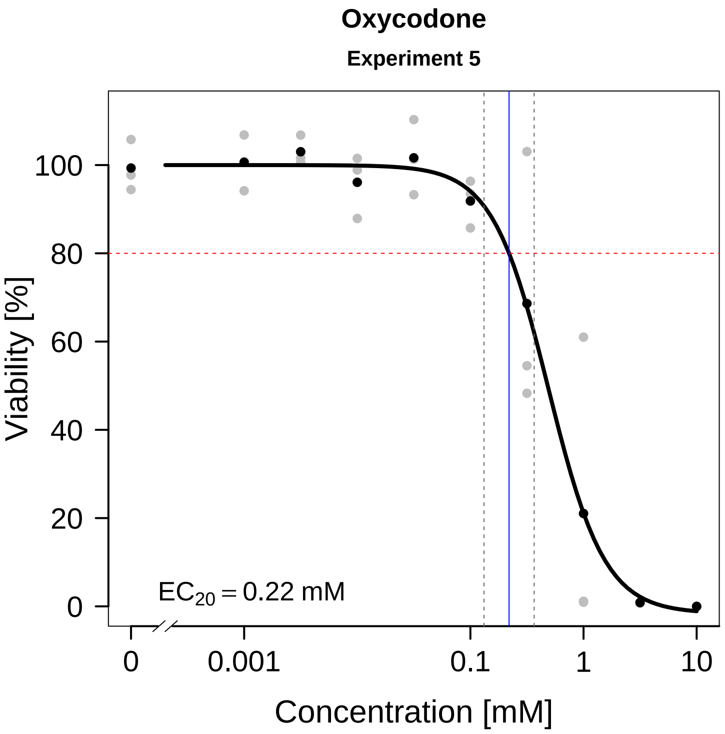
Oxycodone
Experiment 5
Viability [%]
100
80
60
40
20
0
EC20 = 0.22 mM
0
0.001
0.1
1
10
Concentration [mM]

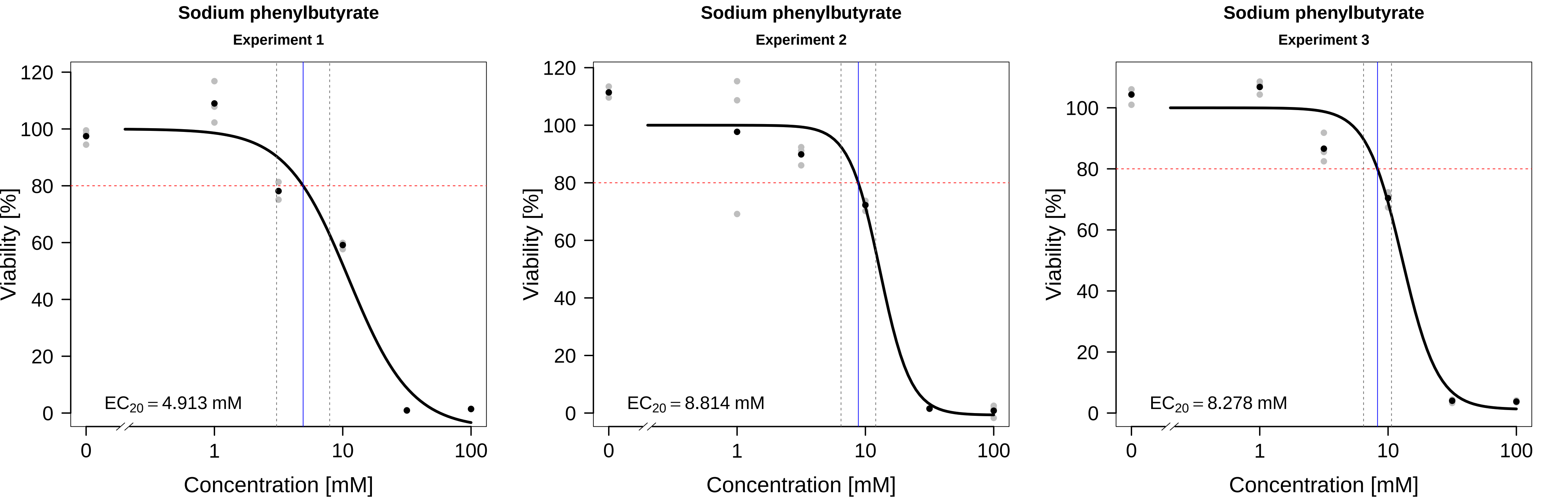

Sodium phenylbutyrate
Experiment 1
Viability [%]
Concentration [mM]
EC20 = 4.913 mM
Sodium phenylbutyrate
Experiment 2
Viability [%]
Concentration [mM]
EC20 = 8.814 mM
Sodium phenylbutyrate
Experiment 3
Viability [%]
Concentration [mM]
EC20 = 8.278 mM

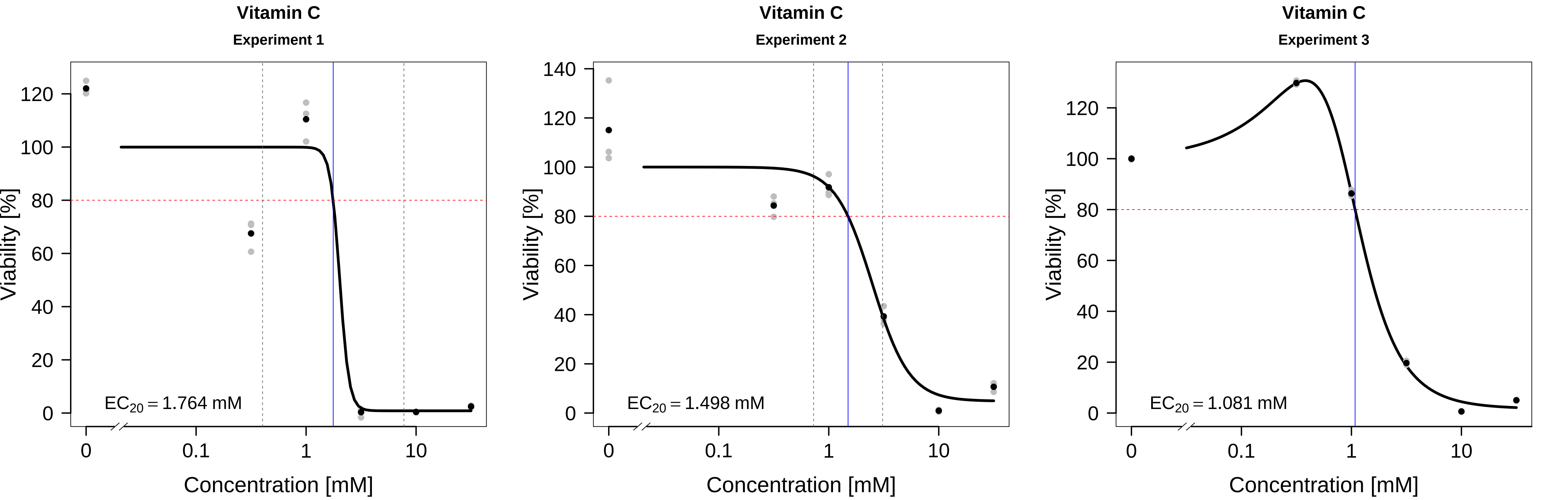
Vitamin C
Experiment 1
EC20 = 1.764 mM
Vitamin C
Experiment 2
EC20 = 1.498 mM
Vitamin C
Experiment 3
EC20 = 1.081 mM
Viability [%]
Concentration [mM]

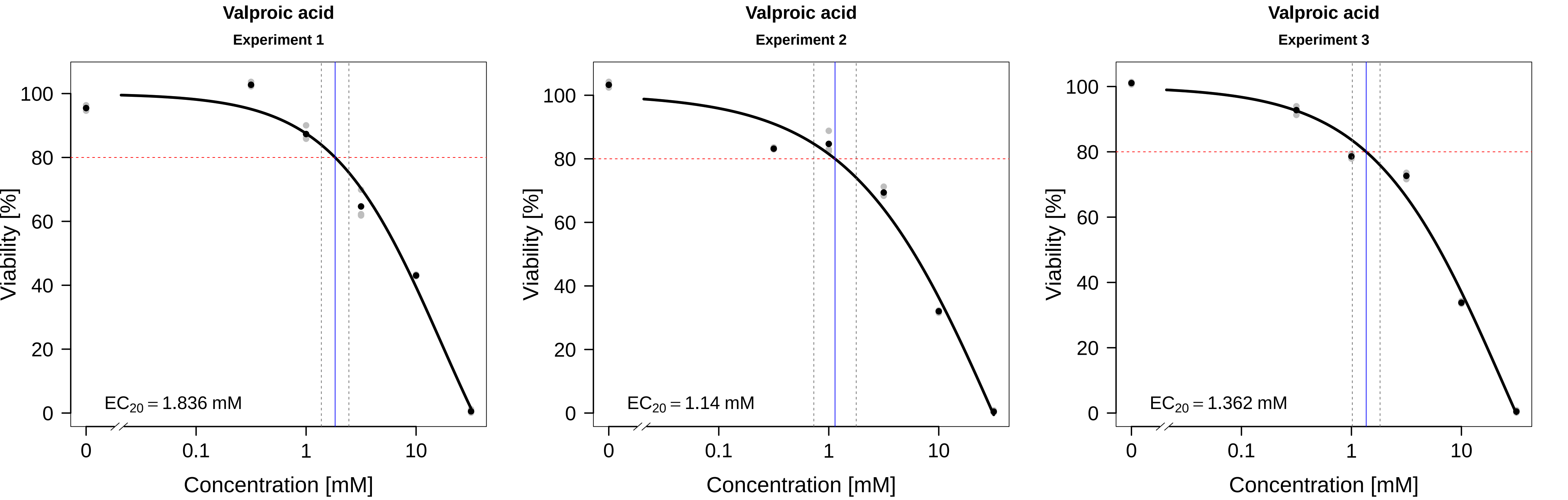

Valproic acid
Experiment 1
Viability [%]
100
80
60
40
20
0
0.1
1
10
Concentration [mM]
EC20 = 1.836 mM
Valproic acid
Experiment 2
EC20 = 1.14 mM
Valproic acid
Experiment 3
EC20 = 1.362 mM
