# Supplementary material for: Adjustment for day-to-day variability in the estimation of effective concentrations for the assessment of mixture toxicity
Source: Arch Toxicol. 2025 Aug 19;99(11):4439–54. doi: 10.1007/s00204-025-04141-w (PMC12477094; doi:10.1007/s00204-025-04141-w)
Supplement: Supplementary file 3 — (pdf 83 KB) [file 204_2025_4141_MOESM3_ESM.pdf]

Supporting Information for 'Adjustment for day-to-day variability in the estimation of effective concentrations for the assessment of mixture toxicity'

Insights on the notation

To emphasize the meaning of the notation introduced within the adjustment approach, this is illustrated here for a fictional example of one substance only. Thus, the index i can be omitted here.

Figure A.1 shows one reference curve (for simplicity, here only one curve is displayed, not several independent reference curves). The fitted parametric curve is denoted as $f(x)$. In the reference curve, the absolute EC$_{20}$ value is calculated as the concentration where the function $f(x)$ intersects with the response value 80%. This value is denoted as c and is formally defined as $c = f^{-1}(x)$, for f^{-1} denoting the inverse function of f. Note: this inverse is only well-defined for the monotonously decreasing 4pLL model. For the non-monotonous BC-model, the EC$_{20}$ is determined via a grid search.

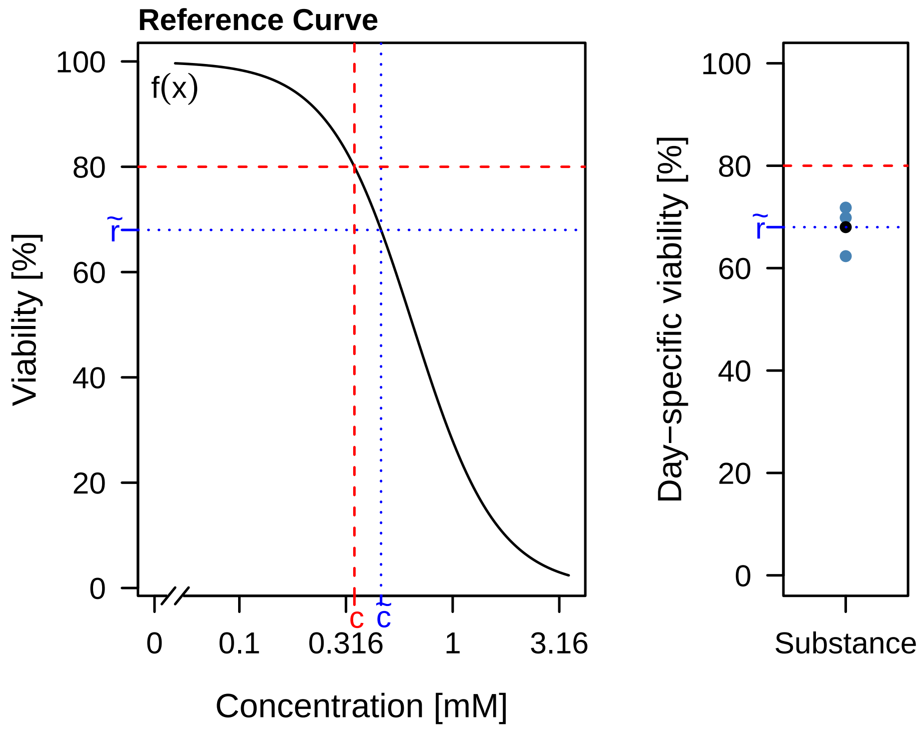

Figure A.1: Emphasis of the relevant notation for the adjustment approach.

The right part of the plot shows the result of the repeated measurement of the viability at concentration c, assessed in this example in three technical replicates (blue dots) at a different day than the reference curve. The mean value (black dot) is the day-specific viability, denoted as $\tilde{r}$.

For the calculation of the adjusted budget, the concentration $\tilde{c}$ in the reference curve is determined, where the curve attains the value $\tilde{r}$, i.e. $f(\tilde{c}) = \tilde{r}$. In the case of f being modelled by a monotonous 4pLL model, this corresponds to defining $\tilde{c}$ as the inverse of f at $\tilde{r}$, i.e. $\tilde{c} = f^{-1}(\tilde{r})$, however, for the BC model, again a grid search needs to be performed.

The adjusted budget for the considered substance is finally calculated as $\frac{\tilde{c}}{c} \cdot 100\%$.
