# Supplementary material for: Adjustment for day-to-day variability in the estimation of effective concentrations for the assessment of mixture toxicity
Source: Arch Toxicol. 2025 Aug 19;99(11):4439–54. doi: 10.1007/s00204-025-04141-w (PMC12477094; doi:10.1007/s00204-025-04141-w)
Supplement: Supplementary file 5 — (docx 430 KB) [file 204_2025_4141_MOESM5_ESM.docx]

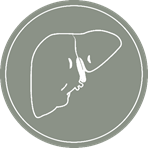

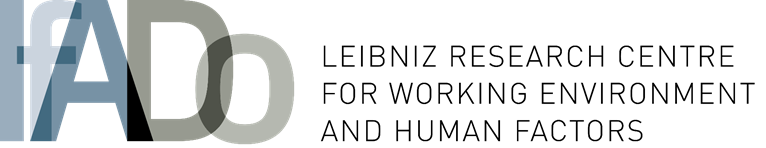


[SOP CTB HepG2]

Date: XX.04.2023

**Contents**

[1 Background 1](#_Toc129879123)

[2 Equipment / consumables 2](#_Toc129879124)

[3 Substances / chemicals 2](#_Toc129879125)

[4 Medium 3](#_Toc129879126)

[4.1 Cultivation medium 3](#_Toc129879127)

[5 Time schedule and general overview 4](#_Toc129879128)

[5.1 Coating 5](#_Toc129879129)

[5.2 Seeding 5](#_Toc129879130)

[5.3 Treatment 5](#_Toc129879131)

[5.4 CTB assay 6](#_Toc129879132)

[6 Statistical analysis 6](#_Toc129879133)

[7 Optional / Comments 7](#_Toc129879134)

#
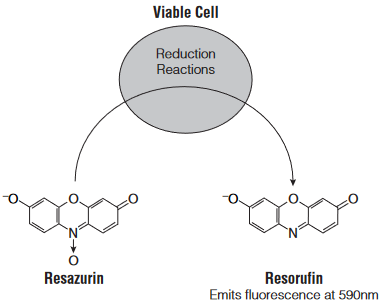
Background*

The CellTiter-Blue® Cell Viability Assay provides a fluorometric method for estimating the number of viable cells present in multiwell plates. It uses the indicator dye resazurin to measure the metabolic capacity of cells—an indicator of cell viability. Viable cells retain the ability to reduce resazurin into resorufin, which is highly fluorescent (Figure 1). Nonviable cells rapidly lose metabolic capacity, do not reduce the indicator dye, and thus do not generate a fluorescent signal.

*Figure 1. Conversion of resazurin to resorufin by metabolically active cells results in the generation of a fluorescent product. The fluorescence produced is proportional to the number of viable cells.*

The CellTiter-Blue® Reagent is a buffered solution containing highly purified resazurin. The ingredients have been optimized for use as a cell viability assay. The spectral properties of CellTiter-Blue® Reagent change upon reduction of resazurin to resorufin (Figure 2). Resazurin is dark blue in color and has little intrinsic fluorescence until it is reduced to resorufin, which is pink and highly fluorescent (579Ex /584Em). The visible light absorbance properties of CellTiter-Blue® Reagent undergo a “blue shift” upon reduction of resazurin to resorufin. The absorbance maximum of resazurin is 605nm and that of resorufin is 573nm. Either fluorescence or absorbance may be used to record results; however, fluorescence is the preferred method because it is more sensitive and involves fewer data calculations.


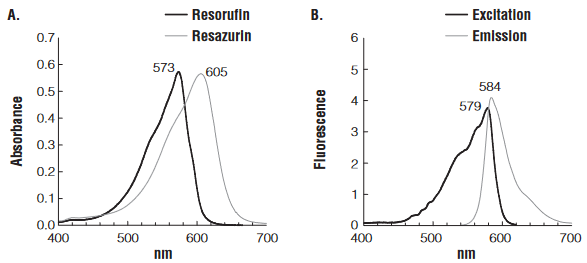


*Figure 2. Spectral properties of resazurin and resorufin in RPMI + 10% fetal bovine serum. Panel A. Absorbance spectra for resazurin and resorufin. Panel B. Fluorescence excitation and emission spectra for resorufin.*

**[Text and Figures copied from CellTiter-Blue® Technical Bulletin, Promega, https://www.promega.de/products/cell-health-assays/cell-viability-and-cytotoxicity-assays/celltiter_blue-cell-viability-assay/?catNum=G8080]*

# Equipment / consumables

- Laminar airflow cabinet
- Incubator (water jacked, 37°C, 5% CO_2_)
- Thermostated water bath or bead bath (37°C)
- Pipet-aid, pipettes and micropipettes, multi-channel pipettes
- Polystyrene tubes (15 mL, 50 mL)
- Microcentrifuge tubes (1.5 mL, 2 mL, 5 mL)
- Ice box
- Paper tissues
- Reagent reservoir
- Black 96-well cell culture plates [e.g. Greiner, 655090]
- Fluorescent plate reader [e.g. Tecan Infinite 200 PRO]

# Substances / chemicals

- Rat collagen lyophilized [e.g. Roche Diagnostic Mannheim, 10 mg]
- 0.2% solution of acetic acid (v/v in dH_2_O, filtered)
- Phosphate buffered saline pH 7.4 (PBS)
- CellTiter-Blue® (CTB) Reagent [Promega, G8081]
- Dulbecco’s Modified Eagle’s Medium (DMEM) [e.g. PAN-Biotech, P04-04500]
- Penicillin-Streptomycin [e.g. PAN-Biotech, P06-07100]
- Fetal Bovine Serum (FBS) *heat inactivated* [e.g. Sera Plus, PAN-Biotech, P30-3702]

*Note: All substances and chemicals should be stored accordingly to the manufactures instructions. Alternatives to the equipment and chemicals listed above can be used if they have the same properties. However, suitability should be verified in a separate pilot test before it is used standardly.*

# Medium

## Cultivation medium

Cultivation medium is used for the cultivation and exposure of cells. It consists of DMEM [PAN-Biotech, P04-04500] with additives as follows:

*Table 1: Medium for cultivation and exposure of HepG2 cells.*

| **Additives** | **For 500 mL medium** | **Final concentration** |
| --- | --- | --- |
| **Penicillin-Streptomycin**  [PAN Biotech, P06-07100] | 5 mL | 100 U/mL Penicillin  0.1 mg/mL Streptomycin |
| **Fetal Bovine Serum (Sera Plus)**  *heat inactivated*  [PAN-Biotech, P30-3702] | 50 mL | 10% |

*Note: Cultivation medium should be consumed within 4-6 weeks. To reduce the contamination or cross contamination risk medium can be aliquoted in 50 mL polystyrene tubes.*

# Time schedule and general overview

HepG2 cells are seeded in black 96-well plates. 15000 living cells per well, as described in the SOP “Cultivation HepG2”. Compound exposure starts the next day after plating. Usually three compounds with five concentrations per compound plus solvent control are tested in three technical replicates per condition on a 96-well plate. Cells are exposed to each test compound for 48 hours. Afterwards, each well is briefly considered under the microscope. Finally, the CellTiter-Blue viability assay is performed. CellTiter-Blue reagent is added to the cells for around 1 hour. After incubation at 37°C the fluorescence intensity is measured at 594 nm using the Tecan Infinite M200 Pro plate reader (i-control software (version 1.7.1.12)).

**
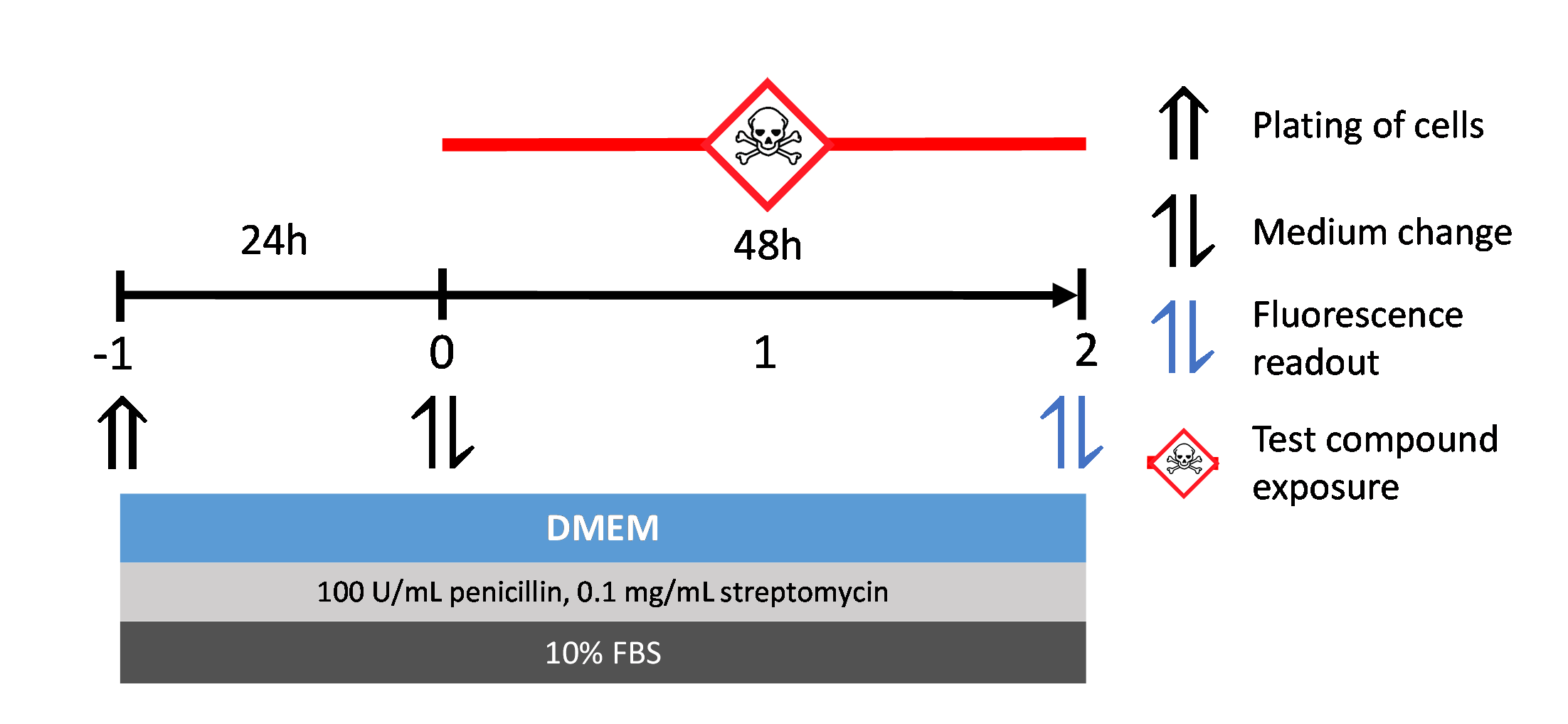
**

*Figure 3: Time schedule and general overview for the CellTiterBlue® cytotoxicity test in cultivated HepG2 cells.*

## Coating

- All steps should be performed under sterile conditions
- Collagen solutions should be constantly kept on ice to prevent gelation process

1. Add 40 mL of 0.2% acetic acid to 10 mg lyophilized collagen (final concentration 0.25 mg/mL) and let it dissolve at least 4 hours in the fridge (+4°C) until no collagen clumps are visible in the solution (dissolving overnight is preferred).
2. Add 100 µL collagen solution per well (96-well plate) and aspirate it back to the stock solution.
3. Leave the plate under the sterile hood overnight to dry.
4. Wash the wells three times with 200 µL 1xPBS before seeding.

*Note: The collagen solution can be reused for up to 4 weeks.*

## Seeding

- All steps should be performed under sterile conditions
- All media should be pre-warmed to room temperature prior use
- Volumes are indicated for T75 flasks

1. Wash the cell culture flask with 10 mL 1xPBS.
2. Add 2 mL trypsin for around 5 minutes at 37°C to detach the cells.
3. Check under the microscope that all cells have detached.
4. Stop the enzymatic process by adding 8 mL cultivation medium.
5. Transfer the cell suspension into a 50 mL polystyrene tube.
6. Centrifuge the cell suspension for 5 minutes at 100 g at room temperature.
7. Discard the supernatant [vacuum pump] and resolve the pellet in 1 mL cultivation medium.
8. Determine the cell number (for example via Trypan Blue exclusion).
9. Seed 15000 cells per well in 200 µL medium and let them rest for 30 minutes under the bench. Afterwards transfer the cells in the incubator (37°C and 5% CO_2_).

*Note: To prevent edge effects the outer wells of the 96-well plate should not be used and just filled with medium or PBS. It was shown that cells attach more uniformly if they are seeded at RT and settle down at RT. If the seeding medium is warmed to 37°C the cell plates should be transferred to the incubator directly.*

## Treatment

Compound exposure starts the next day after plating the cells. Each compound is usually tested in 5 different concentrations (C1 (lowest) to C5 (highest concentration)) plus vehicle controls, 3 wells per condition (technical replicates). Where necessary, the number of concentrations, the dilution factor and the solvent can be selected individually. It is important that the appropriate solvent control is always carried along and that the toxicity of the solvent is tested on the cells beforehand.

- All steps should be performed under sterile conditions
- Corresponding solvent controls should be used

1. Prepare compound solution by:
   1. Using stock solution of a compound dissolved in a solvent [DMSO or EtOH]
   2. Dissolving the compound directly in medium
2. Perform series dilution starting with the highest concentration.
3. Store the solutions in a water bath or bead bath (37°C) for some minutes.
4. Vortex every sample and transfer it to a multi-well reservoir according to the treatment schedule.
5. Collect the plate from the incubator and discard the medium by turning the plate onto a tissue paper. [Make sure that there are as less residues as possible and avoid air bubbles].
6. Add 200 µL of the appropriate treatment solution to each well. [This step should not take longer than 3 minutes. Multi-well reservoirs and multi-channel pipettes are recommended].
7. Place the cells back into the incubator (37°C and 5% CO_2_) and wait for 48 hours.

*Note: The toxicity of solvents has to be tested before usage. Do not exceed a final concentration of 0.5% DMSO or EtOH. Compounds that are dissolved directly in cultivation medium have to be s*terile filtered before starting dilution series.


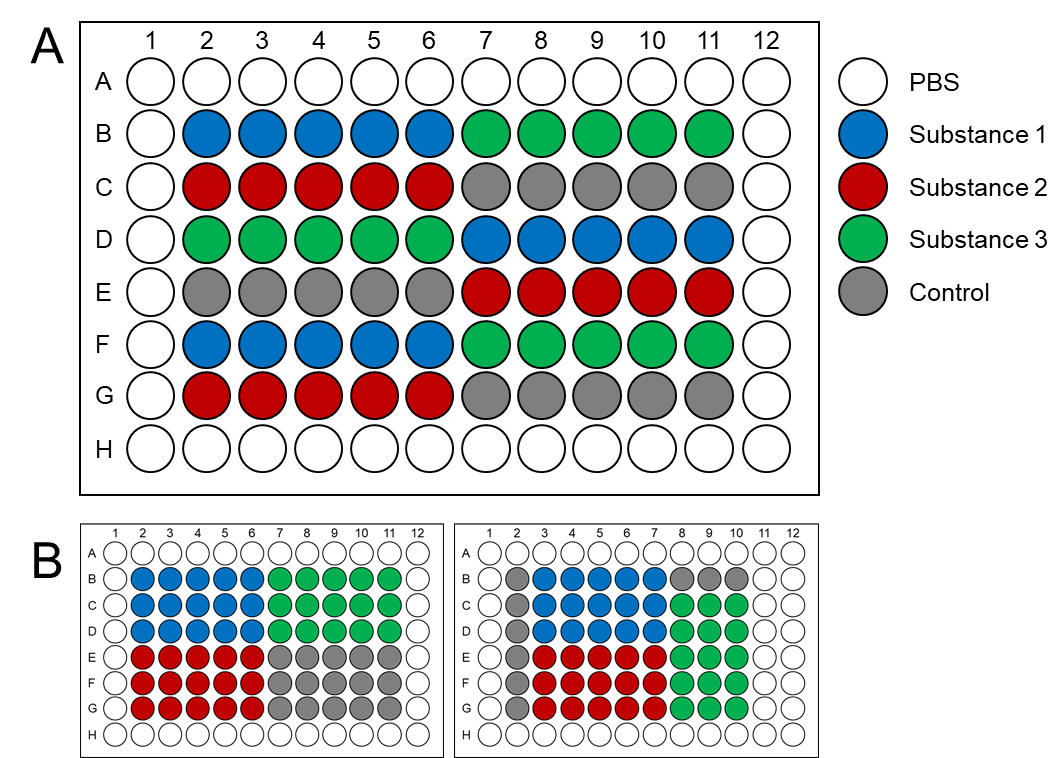


Figure 4: Recommended treatment schedule for 3 substances with 5 concentration and 3 technical replicates (A) and two not recommended schedules (B).

*Note: Since the edge effect can still be measured in the second row, care should be taken to ensure that the individual technical replicates of a concentration or control are evenly distributed over the plate. It is also advisable to distribute the individual concentrations horizontally rather than vertically. In the end, it is up to the experimenter to find a suitable distribution, whereby the edge effect should always be taken into account.*

## CTB assay

- Light should be turned off when working with light-sensitive samples. CTB reagent is light sensitive, therefore the light in the laminar airflow cabinet should be turned off while working with CTB or CTB-medium solution.
- CTB reagent is delivered frozen and should be stored at -20°C. It is recommended to prepare aliquots avoid frequent freezing and thawing.

1. Evaluate the cell morphology for each well under the microscope after 46 - 47 hours of incubation.
2. Place the cells back into the incubator until the 48 hours of exposure are completed.
3. Prepare CTB-medium solution for all plates as follows: Each well requires a total amount of 100 μL fresh CTB-medium solution. 100 μL CTB-medium solution is composed of 80 μL cultivation medium and 20 μL CTB reagent. Prepare a master mix for x+10 wells, you will need (x+10) * 0.08 mL medium and (x+10) * 0.02 mL CTB reagent.
4. Remove the treatment medium after 48 hours by turning the plate upside down on a piece of tissue paper.
5. Wash the cells three times with warm, sterile 1xPBS.
6. Add 100 μL of CTB-medium solution to each well. At least 3 wells without cells should be filled with 100 µL CTB-medium solution as a background control.
7. Place the plate in the incubator for around 1 hour. As soon as a color gradient is visible by eye, the plate can be read out.
8. Use the Tecan Infinite M200 Pro plate reader (software i-control, version 1.7.1.12): Excitation wavelength 540 nm, emission wavelength 594 nm. The output is a fluorescence value for each well of the 96 well plate.

# Statistical analysis

Subtract background values, mean of the wells without cells but CTB-medium reagent. Concentration response curves can be fitted to the data to calculate EC-values.

# Optional / Comments
